# Effectively Regulating More Robust Amorphous Li Clusters for Ultrastable Dendrite‐Free Cycling

**DOI:** 10.1002/advs.202101584

**Published:** 2021-08-03

**Authors:** Shizhi Huang, Junfeng Yang, Luxiang Ma, Jingyi Ding, Xusheng Wang, Chengyuan Peng, Binglu Zhao, Mengxiong Cao, Junrong Zheng, Xin‐Xiang Zhang, Jitao Chen

**Affiliations:** ^1^ Beijing National Laboratory for Molecular Sciences College of Chemistry and Molecular Engineering Peking University Beijing 100871 China; ^2^ Technical Institute of Physics and Chemistry Chinese Academy of Sciences Beijing 100190 China

**Keywords:** amorphous Li clusters, effectively regulating, heteroatom‐activating electronegative sites, orderly multilayer solid electrolyte interphases, ultrastable dendrite‐free cycling

## Abstract

A disordered phase in Li‐deposit nanostructure is greatly attractive, but plagued by the uncontrollable and unstable growth, and the nanoscale characterization in the structure. Here, fully characterized in cryogenic transmission electron microscopy (cryo‐TEM), more robust amorphous‐Li (ALi) clusters are revealed and effectively regulated on heteroatom‐activating electronegative sites and an advanced solid electrolyte interphase (SEI) layer. Heteroatom‐activating electronegative sites capably enhance the electrostatic interaction of Li^+^ and heteroatom‐doping graphene‐like film (HDGs), meaning lower Li diffusion barrier and larger binding energy that is confirmed by small nucleation overpotentials of 13.9 and 10 mV at 0.1 mA cm^−2^ in the fluoroethylene carbonate‐adding ester‐based (FEC‐ester) and LiNO_3_‐adding ether‐based (LiNO_3_‐ether) electrolytes. Orderly multilayer SEI structure comprised of inorganic‐rich components enables fast ion transports and durable capabilities to construct highly reversible and long‐term plating/stripping cycling. ALi cluster anodes exhibit non‐crystalline morphologies and perform ultrastable dendrite‐free cycling over 2800 times. Stable ALi clusters are also grown in LiFePO_4_ (LFP) (LFP‐ALi‐HDGs‐N||LiFePO_4_ [LFP]) full cells with advantageous capacities up to 165.5 and 164.3 mAh g^−1^ in these optimized electrolytes at 0.1 C; the remarkable capacity retentions maintain to 93% and 91% after 150 cycles at 0.2 C. Structure viability, electrochemical reversibility, and excellent performance in ALi clusters are effectively regulated.

## Graphical abstract

Structure viability, electrochemical reversibility, and excellent performance in amorphous Li (ALi) clusters are effectively regulated on the heteroatom‐activating electronegative sites and advanced, multilayer‐ordered, and robust solid electrolyte interphase systems for a more robust ALi cluster anode that exhibits non‐crystalline morphologies and performs ultrastable dendrite‐free cycling over 2800 cycles. Stable ALi clusters are also grown in LFP‐ALi‐HDGs‐N||LiFePO_4_ (LFP) full cells with advantageous capacities.

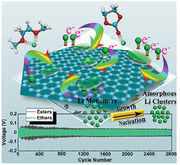

## 1 Introduction

High‐energy‐density and high safety rechargeable batteries are the pursued trends in the growing development of electrochemical energy storage systems.^[^ ^1^, ^2^, ^3^, ^4^ ^]^ Lithium metal materials, owning a theoretical specific capacity of up to 3860 mAh g^−1^ and an electrochemical potential as low as −3.04 V (vs the standard hydrogen electrode), are recognized as the ideal electrode materials in next‐generation high‐performance lithium‐ion batteries.^[^ ^5^, ^6^ ^]^ However, several vital issues in the Li‐metal anodes including crystal‐type growth of high‐risk lithium dendrites, extremely unstable solid electrolyte interphase (SEI), and deteriorative volume variation in Li plating/stripping, severely restrict its industrial application.^[^ ^5^, ^6^, ^7^ ^]^ Li dendrites growing with high crystal orientation would penetrate into the separators and cause a short circuit in the batteries, which would induce serious safety hazards.^[^ ^8^, ^9^ ^]^ During Li deposition/exfoliation, the volume expansion is uncontrollable, resulting in the breakages of unstable SEI layer, as well as the Coulomb efficiency and reversible capacity decaying closely with the repeated fractures of SEI and continuous electrolyte consumption.^[^ ^6^ ^]^ In addition, isolated Li‐dendrites are formed after cycling and gradually turn into the dead Li, which easily accumulated on the electrode surfaces and intensified the polarization of Li electrodes.^[^ ^6^, ^10^, ^11^, ^12^ ^]^

Recently, based on the research hotspots of lithium metal challenges, electrolyte modification,^[^ ^13^, ^14^, ^15^ ^]^ SEI stabilization design,^[^ ^16^, ^17^, ^18^, ^19^, ^20^ ^]^ solid electrolyte engineering,^[^ ^21^, ^22^, ^23^ ^]^ three‐dimensional (3D) structured Li electrodes,^[^ ^24^, ^25^, ^26^, ^27^ ^]^ lithiophilic nucleation seeds,^[^ ^26^, ^28^ ^]^ high‐strength separators,^[^ ^29^, ^30^, ^31^ ^]^ etc., are extensively demonstrated. But among them, the Li‐phase evolution at the nanoscale during the nucleation and growth has been rarely reported. Liaw et al. successfully used cryogenic transmission electron microscopy (cryo‐TEM) to reveal a disorder–order phase evolution in Li‐nucleation and growth through the control of current density and deposition time.^[^ ^32^ ^]^ An amorphous crystal structure in the Li‐deposit nanostructure definitely appears with a disordered crystal orientation, and avoids the Li‐dendrite formation in high crystallization. However, the stability regulation of plating amorphous Li clusters remains uncontrollable and elusive, and greatly relies on the Li plating matrixes and SEI optimization. Depositing Li on the lithiophilic 3D conductive skeletons, especially the 3D graphene‐based conductive carbonaceous materials, can effectively reduce local current density through a high specific surface area.^[^ ^7^, ^10^, ^33^ ^]^ Simultaneously, the lithiophilic 3D conductive skeletons preferably accommodate to the volume expansion.^[^ ^34^, ^35^, ^36^ ^]^ Furthermore, introducing heteroatoms into the lattices of carbonaceous materials, such as nitrogen‐doped patterns, can form the heteroatom‐activating electronegative sites and offer lower nucleation energy barrier and larger binding energy by a more desirable lithiophilicity to regulate homogeneous Li nucleation and growth (Table S1, Supporting Information).^[^ ^22^, ^34^, ^37^ ^]^ Apart from the construction of 3D conductive skeletons, stabilizing SEI has aroused wide concern for the protection of Li‐metal anodes, in particularly building a robust inorganic‐rich SEI with high interface energy.^[^ ^6^, ^18^, ^38^ ^]^ Wang and coworkers have prepared a high‐interface‐energy LiN*_x_* O*_y_*‐LiF‐rich SEI using LiNO_3_ as an additive to accelerate the Li diffusion on the SEI/Li interface and construct a strong structural protection, which capably alleviate the Li‐dendrite growth and penetration.^[^ ^17^ ^]^ Fluoroethylene carbonate (FEC) added to the carbonate electrolytes can also conduct a well‐ordered multilayer SEI structure composing of inorganic and organic components for improving Li electrode performance.^[^ ^18^, ^39^, ^40^ ^]^

Herein, heteroatom‐doping electronegative sites arising from F, O, N, and Cl codoping in the graphene lattices, are developed for regulating amorphous‐Li (ALi) cluster plating on HDGs. With the effective characterization of cryo‐TEM, the structurally stable ALi clusters are presented. A high surface area of 1764 m^2^ g^−1^ and lithiophilic surface on the HDGs contribute much sense for achieving a viable and efficient amorphous Li‐metal anode. The local current density can be effectively reduced by the high‐surface‐area HDGs plating matrix through a high surface area. As referenced in Table S1, Supporting Information, the results of the first principles calculations, F, O, N, and Cl activating heteroatom‐sites significantly promote the binding energy between doping heteroatoms and a Li atom, so these electronegative sites generated by the heteroatom doping could enhance the Li^+^/HDGs electrostatic interaction. Therefore, Li ions would be rapidly driven to these lithiophilic sites, and then adsorbed and reduced on the HDGs activating surface. Subsequently, Li starts to nucleate and performs small nucleation overpotentials of 13.9 and 10 mV at 0.1 mA cm^−2^ in the fluoroethylene carbonate‐adding ester‐based (FEC‐ester) and LiNO_3_‐adding ether‐based (LiNO_3_‐ether) electrolytes, respectively, which reveals a low Li nucleation barrier and a large binding energy. Moreover, to further improve the structural stability of ALi‐HDGs, FEC and lithium nitrate (LiNO_3_) additives are, respectively, introduced into ester and ether electrolytes to construct orderly multilayer SEI of inorganic‐rich components. The ordered multilayer structure and inorganic‐rich interface in the SEI can increase the Li ion transports and mechanical abilities, and thus, the ALi anodes stably conduct plating/stripping processes over 2800 cycles, as well as achieving high Coulombic efficiency (CE) of >99% on average. The LFP‐ALi‐HDGs‐N||LiFePO_4_ (LFP) full cells are successfully constructed, ALi clusters can also grow, and it performs excellent properties up to 165.5 and 164.3 mAh g^−1^ at 0.1 C in the FEC‐ester and LiNO_3_‐ether electrolytes. The capacity retention rates at 0.2 C cycling are up to 93% and 91% after 150 cycles.

## 2 Results and Discussion

### 2.1 Effectively Regulating Mechanism for Amorphous‐Li Clusters Plating on the Heteroatom‐Activating Electronegative Sites

**Figure 1:**
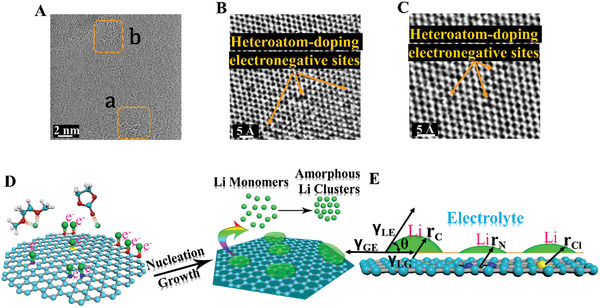
A–C) HAADF‐STEM images of HDGs plating matrixes in atomic‐scale; (B) and (C) corresponded to the marked regions a and b in (A). D) Regulating mechanism of the ALi clusters on the heteroatom‐activating electronegative sites. E) Li nucleation on different plating matrixes based on the classical heterogeneous nucleation mode.

Lithiophilic 3D HDGs plating matrixes in possession of hierarchical open‐type porous structure have been prepared via K‐activated etching methods (Figure S1, Supporting Information). In the pyrolytic graphitization process, the generating K compounds (Equations S1–S5, Supporting Information) acted as the activating reagents and positively etched the carbon framework in resins to produce 3D ultrathin graphene sheets that assembled a hierarchical open‐type porous structure.^[^ ^41^ ^]^ H_2_, CO, CO_2_, and vaporized H_2_O formed in the pyrolysis decomposing also contribute to the construction of the 3D hierarchical open‐type porous structure through gasification and gas interaction.^[^ ^41^, ^42^, ^43^ ^]^ Meanwhile, the thermal decompositions of the ethylenediamine curing agents, triethanolamine accelerators and epichlorohydrin intermediates in the fluorinated epoxy resins result in the codoping of F, O, N, and Cl into HDGs graphene lattices, making it preferable with heteroatom activating defects as the heteroatom‐doping electronegative sites. As shown in **Figure** **1**A–C, the atomic‐scale characterizations of these heteroatomic sites are evidently formed in HDGs graphene lattices. Apparently, irregular membered rings consisting of the heteroatom introduction in carbon‐atom membered rings, especially the five‐membered rings or their multiple combinations appear exactly in the lattice defects and remain stable as the heteroatom‐activating electronegative sites. In X‐ray photoelectron spectroscopy (XPS) measurements (Figures S2–S4, Supporting Information), F, O, N, and Cl are affirmatively doped into the graphene lattices, which could produce abundant electrochemical activating sites for regulating the ALi nucleation and growth. In the Raman spectra (Figure S5, Supporting Information), the characteristic D peak at 1358 cm^−1^ further reveals the presence of heteroatom‐doping defects as the electronegative sites in the graphene lattices.^[^ ^44^ ^]^ In Table S1, Supporting Information, as referenced to the results of the first principles calculations, O‐ and N‐type dopants in the conductive carbonaceous materials as the activating heteroatom‐sites present larger binding energy between doping heteroatoms and a Li atom. The larger binding energies of −4.26, −4.46, −3.46, −2.86, −2.35, and −1.91 eV are corresponded to the pyridinic N, pyrrolic N, graphitic N, carboxylic group O, ketone group O, and hydroxyl group O, which also appeared in this HDGs plating matrix material.^[^ ^22^, ^34^, ^37^ ^]^ The F and Cl dopants also enhance the surface properties for the sensitive adsorption of Li atoms to these dopant sites.

High specific surface area of up to 1764 m^2^ g^−1^ and preferable electrical conductivity of ∼829.87 S m^−1^ on the HDGs plating matrix, that determined by a BET test and a four‐probe method, can effectively reduce local current density, that heavily affecting the ALi nuclei (Figure S6, Supporting Information).^[^ ^37^, ^45^ ^]^ A referred quantitative model used to determine the local current density is as follows:^[^ ^46^ ^]^ the specific surface area of the HDGs plating matrix is 1764 m^2^ g^−1^ and an areal loading weight of HDGs materials on each Cu piece is ∼1.2 mg cm^−2^. Thus, the local surface area of HDGs plating matrix is 2.12 m^2^ per 1.0 cm^−2^. In our research, a current density of 0.1 mA ${\mathrm{cm}}_{\mathrm{Cu}\;\mathrm{piece}}^{-2}$ is cycled and an ultralow local current density of Li deposition on HDGs plating matrix is calculated as 4.72 × 10^−6^ mA ${\mathrm{cm}}_{\mathrm{HDGs}}^{-2}$, that is 4.72 × 10^−5^ times of the Cu foil (0.1 mA ${\mathrm{cm}}_{\mathrm{Cu}\;\mathrm{piece}}^{-2}$).

Precisely regulated by the surface modification of heteroatom activating sites, electrostatic interaction between the solvated Li^+^ and the heteroatom‐doping electronegative sites would be greatly promoted, so the Li ions can be rapidly driven to the lithiophilic sites, and then adsorbed and reduced (Figure 1D). After agglomerating to a certain size, the reduced Li atoms on the activating HDGs matrixes start to nucleate and exhibit small nucleation overpotentials of 13.9 and 10 mV at 0.1 mA cm^−2^ in the ester‐ and ether‐electrolytes (shown in Figure S7, Supporting Information). It reveals a lower Li nucleation barrier, and indicates a larger binding energy for Li nucleating on the heteroatom‐activating electronegative sites. According to the classical heterogeneous nucleation theory, as illustrated in Figure 1E and Equations S6–S9, Supporting Information, the critical Li‐nucleation radius (r*) is fixed, but the lower Li nucleation barrier would reduce the needed volume of Li‐nucleation in reaching r*.^[^ ^37^ ^]^ Due to the large binding energy on the heteroatom‐activating electronegative sites, Li would nucleate and grow in a small volume to obtain the ALi clusters due to the aggregation of a certain amount of Li atoms in a disorder pattern. HDGs plating matrixes with a high surface area and heteroatom‐activating surface of abundant electronegative sites conduct a positive significance for regulating a stable and efficient ALi cluster anodes.

### 2.2 Cryogenic Transmission Electron Microscopy Characterizations on Amorphous‐Li Clusters

**Figure 2:**
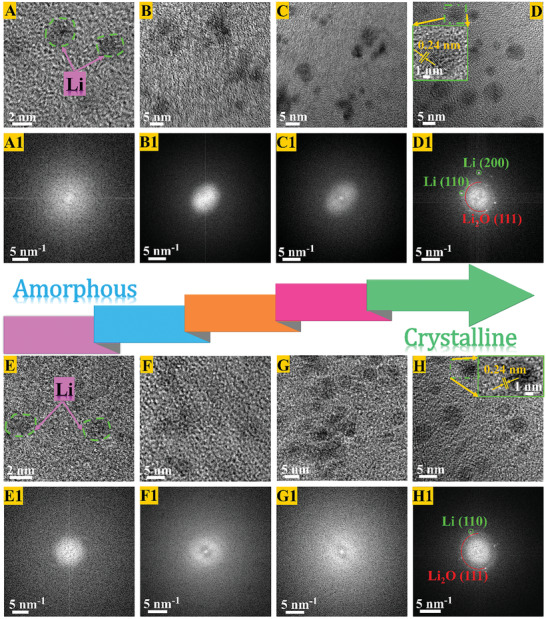
Cryo‐TEM characterizations on disorder–order evolving nanostructures of Li plating on activating HDGs matrixes. A–H) High‐resolution cryo‐TEM images and A1–H1) their corresponding FFT patterns of Li plating at 0.1 mA cm^−2^. The nucleation (A,E,A1,E1), and the growths for 10 min (B,F,B1,F1), 20 min (C,G,C1,G1), and 30 min (D,H,D1,H1) in FEC‐ester electrolytes (A–D,A1–D1) and LiNO_3_‐ether electrolytes (E–H,E1–H1). The nanostructure evolutions of Li deposits correspond to the deposition voltage/time curves in Figure S7, Supporting Information.

Disorder–order evolving nanostructures of ALi clusters plating on heteroatom‐activating electronegative sites were characterized on cryo‐TEM in **Figure** **2**. At the cryogenic conditions (∼−176 °C), the holed damages caused by electron beam irradiation can be effectively minimized, and makes ALi clusters show detailed nanostructures and its SEI more stable.^[^ ^47^, ^48^ ^]^ At 0.1 mA cm^−2^ (Figure S7, Supporting Information), as the deposition time increases, the reduced Li atoms adsorbed on the HDGs activating sites would aggregate together and start to nucleate with a small overpotential as the driving force. As characterized in Figure 2A,E, nanocluster microstructures in a small size of ∼2 nm are revealed in the ALi nucleation, and no obvious lattice fringes appear. There are also no characteristic body‐centered cubic bright diffraction spots/rings of Li metal in fast Fourier transformed (FFT) images (Figure 2A1,E1), which suggests that the phase types in nucleation of ALi deposition are amorphous or glassy in FEC‐ester and LiNO_3_‐ether electrolytes. After nucleating, either apparent Li lattice fringes (Figures 2B,C and 2F,G) or distinct Li diffraction spots/rings in FFT images (Figures 2B1,C1 and 2F1,G1) is detected and even the deposition evolution of Li extends and develops to 10 and 20 min. It suggests that larger ALi clusters growing with sizes of 3–5 nm are determined to amorphous structure on the HDGs activating matrixes. The areal capacities of as‐prepared ALi that are plated at 0.1 mA cm^−2^ for 10 and 20 min to maintain the amorphous structure are ∼0.017 and 0.033 mAh cm^−2^ (Figure S7, Supporting Information).

In Figure 2D,H, a portion of ALi clusters stabilized on the HDGs activating surface have gradually crystallized and presented characteristic lattice fringes with a 0.24 nm spacing distance between two adjacent lattice fringes, that is consistent with the Li (110) lattice planes.^[^ ^32^, ^49^ ^]^ Meanwhile, crystalline Li diffraction spots in FFT images also appear, that indicates the formation of crystalline Li in Li (110) and Li (200) lattice planes (Figure 2D1,H1).^[^ ^49^ ^]^ The red marked diffraction rings in FFT images are related to the Li_2_O (111) lattice planes, suggesting a part of inorganic components in the SEI. In general, as the deposition‐time increases, more Li atoms have been reduced and larger ALi clusters have grown and stabilized on the activating HDGs matrixes that effectively regulates on the heteroatom‐activating electronegative sites. According to the classical heterogeneous nucleation theory, as illustrated in Figure 1E and Equations S6–S9, Supporting Information, the nuclei of Li will appear when its bulk energy overcomes the surface energy, and later when the embryo size exceeds the critical radius, the Li nuclei will grow and the ALi clusters are formed.^[^ ^37^ ^]^ In this electrochemical deposition, these mentioned processes are driven by the charge transfer and the mass transport of Li ions near the electrolyte–electrode interface. With the deposition time increases, more reduced Li aggregates and maintains a supersaturation on the surface of ALi by the galvanostatic driving forces. Actually, these ALi clusters are the transition phases, which are metastable and have high bulk energy. Last, a supersaturation of mass transfer and the bulk energy reduced trend can drive the disordered ALi into the ordered crystalline Li, that is shown in the Figure 2D,H.^[^ ^50^, ^51^ ^]^ Moreover, the atomic interaction in the nucleation and growth process can also promote the crystalline transformation.^[^ ^32^ ^]^

**Figure 3:**
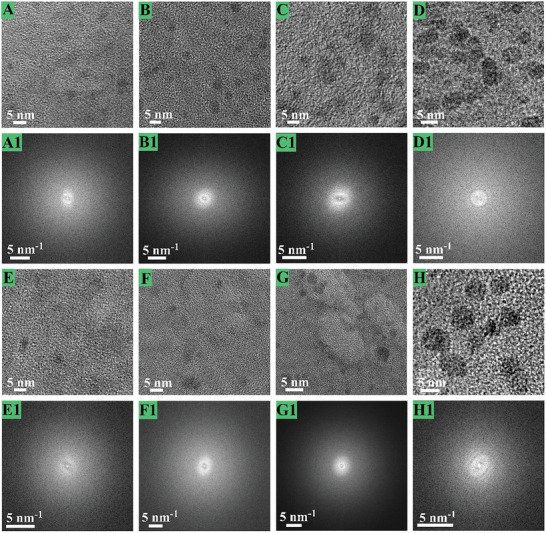
High‐resolution cryo‐TEM images and their corresponding FFT patterns of ALi cluster nanostructures. ALi clusters are deposited on activating HDGs matrixes at increased current densities of A,A1,E,E1) 0.2, B,B1,F,F1) 0.5, C,C1,G,G1) 1 and D,D1,H,H1) 2 mA cm^−2^ for growing 2 s after nucleation. The electrolytes are FEC‐ester electrolytes (A–D,A1–D1), and LiNO_3_‐ether electrolytes (E–H,E1–H1).

To confirm the effectiveness of ALi clusters growing at increased current densities (Figure S8, Supporting Information), further researches of the ALi plating on the HDGs activating matrixes in FEC‐ester electrolytes and LiNO_3_‐ether electrolytes at 0.2, 0.5, 1 and 2 mA cm^−2^ have been conducted. In the high‐resolution cryo‐TEM characterizations (**Figure** **3**), Li was deposited on the HDGs activating surfaces for growing 2 s after nucleating; the nanoscale clusters possessing a desired disorder structure have been proved. Even in the corresponding FFT images, no relevant information about the crystalline Li structures is observed. The size domains of these ALi clusters are 2–5 nm that stays firmly on the activating HDGs matrixes. Obviously, higher current densities would produce larger nanoscale ALi clusters. For instance, ALi clusters of ∼5 nm are obtained at 2 mA cm^−2^; the ALi cluster sizes strongly depend on the applied current densities. As a result, increasing the current densities would result in a wider distribution of ALi clusters; this is because, applying higher current densities would increase the electron fluxes and accelerate the amounts of reduced Li to deposit as larger ALi clusters on the surface of activating HDGs matrixes. Additionally, almost identical results are obtained in FEC‐ester and LiNO_3_‐ether electrolytes. The super lithiophilicity nature of these HDGs plating matrixes is shown in Figure S9, Supporting Information; the small nucleation overpotentials at the various current densities are measured. Although the current densities enlarge 20 times to 2 mA cm^−2^, only a small nucleation overpotential of 2.7 times is detected.

### 2.3 An Advanced Solid Electrolyte Interphase System on ALi‐HDGs

**Figure 4:**
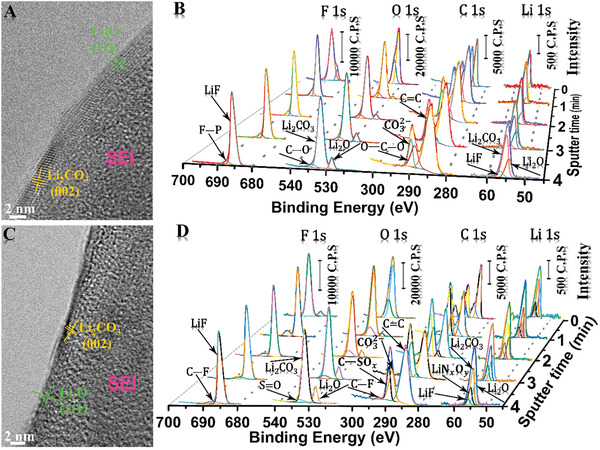
Advanced, orderly, and robust SEI systems. High‐resolution cryo‐TEM images and their corresponding in‐depth XPS measurements of SEI formed on ALi‐HDGs anodes in A,B) FEC‐ester electrolytes and C,D) LiNO_3_‐ether electrolytes.

An advanced, orderly, and robust SEI system on ALi‐HDGs is highly required for stabilizing ALi clusters in the plating/stripping cycling. Herein, we have, respectively, prepared 13 wt% FEC and 0.2 m LiNO_3_ additives into ester and ether electrolytes to construct an excellent multilayer‐ordered SEI of inorganic‐rich components (**Figure** **4** and Figures S10–S17, Supporting Information). The electrochemical redox behaviors of SEI formation were investigated by the cyclic voltammetry (shown in Figure S10, Supporting Information). In Figure S10A, Supporting Information, it clearly suggests that the first reduced peak of FEC containing additive is observed at 1.31 V, demonstrating that the reductive decomposition of FEC tends to be intense.^[^ ^52^, ^53^ ^]^ Besides, a wide peak attributed to the decomposition of LiPF_6_/ethylene carbonate (EC)/dimethyl carbonate (DMC) appears at 0.3–0.8 V and it fully proves a sequential decomposition process of SEI forming in the FEC‐ester electrolytes.^[^ ^53^ ^]^ Meanwhile, in the LiNO_3_‐ether electrolytes, ${\mathrm{NO}}_{3}^{-}$ is reduced at 1.69 V, and the decomposition peak of TFSI^−^ presents at 1.49 V. The next reduction of DME/DOL occurs at ≈0.75 V (Figure S10B, Supporting Information).^[^ ^17^ ^]^ It also leads to a well‐ordered decomposition in differentiated voltage range for SEI ordered forming. FEC and LiNO_3_ additives contribute to enabling a robust inorganic‐rich SEI, especially the LiF‐rich forming in the SEI components. FEC has a lower lowest unoccupied molecular orbital than the traditional carbonate electrolytes, which preferentially reduces and facilitates the LiF‐rich generation in SEI.^[^ ^40^ ^]^ As for LiNO_3_, the presences of ${\mathrm{NO}}_{3}^{-}$ capably promote the interactions of TFSI^−^and Li^+^ by participating in the solvation sheath, which is conducive to LiN*_x_* O*_y_*‐LiF‐rich SEI formation.^[^ ^17^ ^]^ More importantly, the characteristic peaks mentioned above disappear in the next four cycles, and it means that the SEI forming structure gradually becomes stable (Figure S10C,D, Supporting Information). FEC and LiNO_3_ additives really impact the stability of SEI on ALi‐HDGs. In high‐resolution cryo‐TEM images (Figure 4A,C), similar growths of excellent multilayer‐ordered structures are apparently formed in FEC‐ester electrolytes and LiNO_3_‐ether electrolytes. These include crystalline domains completely covered on the outer layers and amorphous polymer matrixes appearing in the inner sides. The crystalline domains of outer layers are determined to the Li_2_O and Li_2_CO_3_ by the lattice spacing measurements (Figures S11–S14, Supporting Information).^[^ ^26^, ^47^ ^]^

Further investigations of SEI composition on in‐depth XPS characterization by the Ar^+^ sputtering for 0, 1, 2, 3, and 4 min are measured in Figure 4B,D, as well as in Figures S15–S17, Supporting Information. In Figure 4B, the SEI formed in FEC‐ester electrolytes, the intensities, and widths of LiF peaks in F 1s are intense all throughout the depth tests, while the Li_2_O peak intensities in O 1s gradually increase during the Ar^+^ sputtering process.^[^ ^54^ ^]^ It implies that a thicker LiF layer was formed. The measured results of LiF, Li_2_CO_3_, and Li_2_O in C 1s and Li 1s also totally exhibit the similar trends.^[^ ^39^ ^]^ In P 2p spectra, a significant fluorine‐rich fluoro‐polyphosphate (Li*_x_* PO*_y_*_−1_F*_z_*_+1_) tends to grow on outer surface that is validated by the single signal in Figure S15F, Supporting Information, and then is gradually weakened in the next Ar^+^ depth sputtering (Figure S15G–J, Supporting Information). Overall, high‐content FEC additive introduced in ester electrolytes facilitates the forming in inorganic‐rich species, particularly the F‐containing components.^[^ ^55^, ^56^ ^]^ In ether‐type electrolytes (Figure 4D and Figures S16 and S17, Supporting Information), the LiF peaks and widths in F 1s also maintain constant during the whole Ar^+^ sputtering process, which indicates a deeper LiF layer formed in high contents. Meanwhile, the signals of Li_2_O in O 1s continue to increase, displaying the same result in the ester electrolyte system. Moreover, the N‐containing LiNO_2_ and LiN*_x_* O*_y_* species from LiNO_3_ decomposition are revealed in Figure S17, Supporting Information. It fully evidences that the LiNO_3_ additive in ether‐type electrolytes can form a deeper inorganic layer, which is rich in the multilayer‐ordered SEI. An orderly decomposition process in differentiated voltage range could be considered the primary reason for forming multilayer ordered SEI. At the certain regular potentials, the SEI generated species can be discriminatively homogenized and arranged at a separable voltage. Inorganic‐rich species can promote interfacial stability, especially the LiF with high interface energy is conducive to adapt to the volume variation of ALi‐HDGs anode, while the capabilities of ordered multilayer structure can enhance the mechanical feasibilities, optimize the ion diffusion paths, and perform a durable cycling.^[^ ^57^, ^58^, ^59^, ^60^ ^]^ These uniform and rapid Li ion diffusion on this advanced, orderly, and robust SEI contribute to sustain enough mass transport of Li ions near the electrolyte–electrode interface, and the homogeneous Li depositions can be obtained.^[^ ^61^, ^62^, ^63^ ^]^ This SEI is smooth and has high mechanical strength, ensuring effective improvements of the electrode reversibility, interface stability, and uniformity of Li deposition. Thus, an ultrastable dendrite‐free deposition of homogeneous ALi is effectively regulated on this advanced, multilayer‐ordered, and robust SEI for a more robust ALi cluster anode.

### 2.4 Electrochemical Performance on Amorphous‐Li Cluster Anodes

**Figure 5:**
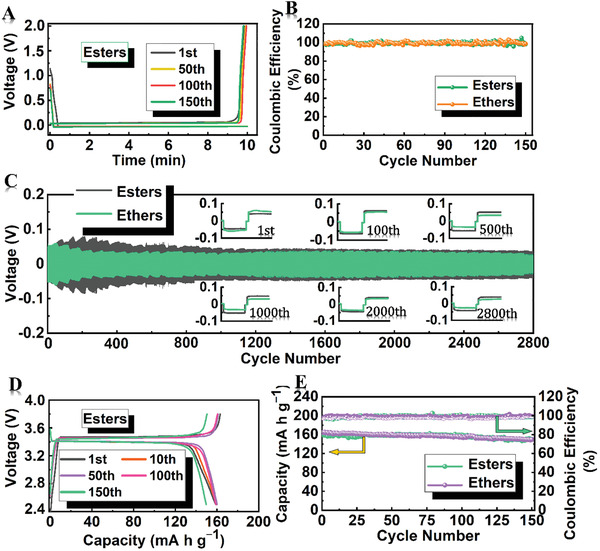
Electrochemical performance of ALi‐HDGs anodes in FEC‐ester electrolytes and LiNO_3_‐ether electrolytes. A) Voltage–time profiles and B) Coulombic efficiency of Li plating/stripping at 0.1 mA cm^−2^ for 10 min. C) Symmetrical cell performance of ALi‐HDGs‐10 plating/stripping at 0.1 mA cm^−2^ for 10 min in each cycle. D) Charging/discharging profiles in FEC‐ester electrolytes and E) their corresponding cycling properties of ALi‐HDGs‐N||LiFePO_4_ (LFP) full cells at 0.2 C.

Before Coulombic efficiency (CE) measuring in ALi cluster anodes, the activating HDGs matrixes were cycled at 0.1 mA cm^−2^ for ten cycles between 3 and 0.01 V for SEI stabilizing. Li was plated at 0.1 mA cm^−2^ for 10 or 20 min to obtain amorphous structures in the plating/stripping process, and the stripping voltage was 2 V (**Figure** **5**A,B and Figures S18 and S19, Supporting Information). Highly initial CEs of 98.7% and 98.4% were measured at 0.1 mA cm^−2^ for 10‐min plating/stripping in FEC‐ester electrolytes and LiNO_3_‐ether electrolytes. And after 150 cycles, all these CEs remain at ≈99.3%. Even the plating/stripping increases to 20 min at 0.1 mA cm^−2^ and the CEs are maintained ∼99% after 100 cycles (Figure S19, Supporting Information). More homogeneous ALi deposition on the HDGs plating matrixes forms a smooth Li surface and the orderly multilayer structure comprised of inorganic‐rich components in an advanced SEI enables fast Li ion transports, also suggesting a highly reversible Li stripping. Such high CEs demonstrate that the ALi‐HDGs anodes have highly reversible plated/stripped processes in this feasible ALi structure. In Figure 5C, plating/stripping stabilities of ALi clusters were further examined in the symmetric cells with Li plating at 0.1 mA cm^−2^ for 10 min (ALi‐HDGs‐10). As cycles proceed at 0.1 mA cm^−2^ for plating/stripping 10 min, the ALi‐HDGs‐10 anodes steadily exhibit lower polarization that can be stabilized at ∼37 and ∼33 mV in the FEC‐ester electrolytes and LiNO_3_‐ether electrolytes. After 1000 plating/stripping cycles, both in FEC‐ester electrolytes and LiNO_3_‐ether electrolytes, the cycled ALi clusters well preserve on the activating HDGs matrixes (Figures S20 and S21, Supporting Information). Furthermore, long‐term cycling of more than 2800 cycles is evidently illustrated and the excellent electrochemical cycling stabilities are achieved. It strongly proves that lithiophilic activating‐surface and an advanced, orderly, and robust SEI system on activating HDGs plating matrixes composed of heteroatom‐activating electronegative sites are conducive to the effective regulation for stabilizing ALi clusters.

To further demonstrate the regulated effect of ALi clusters stably growing on the activating HDGs plating matrixes, LFP and SEI‐stabilized ALi‐HDGs‐N were used as the Li sources and Li hosts, respectively. With Li deposition from LFP cathode, it can verify the specific depositing states of Li growing in the practical batteries, that can compare it with the ALi growths from pure Li pieces in synchronization. After LFP charging to 3.8 V at 0.1 C, ALi cluster growths on activating HDGs hosts (LFP‐ALi‐HDGs‐N) of ∼5 nm are also observed in Figures S22 and S23, Supporting Information, and are identical to the ALi clusters from pure Li deposition. The stably amorphous nanostructure can endow a definitely dendrite‐free anode for LFP cycling in the LFP‐ALi‐HDGs‐N||LFP full cells, and making it away from the safety hazards caused by the Li‐dendrites during the cycling. Highly initial specific capacities of 165.5 and 164.3 mAh g^−1^ in Figure S24A,B, Supporting Information, are obtained at 0.1 C in FEC‐ester electrolytes and LiNO_3_‐ether electrolytes with over 99.1% CEs after 80 cycles (in Figure S24C, Supporting Information). At 0.2 C cycling in Figure 5D,E, advantageous capacities are also achieved up to 159.3 and 164.4 mAh g^−1^ in FEC‐ester electrolytes and LiNO_3_‐ether electrolytes, including the remarkable capacity retentions of 93% and 91% after 150 cycles (in Figure 5D and Figure S25, Supporting Information). It suggests that these more robust ALi cluster anodes during the cycling enable stable dendrite‐free cycling, highly reversible processes, and excellent electrochemical performance in LFP‐ALi‐HDGs‐N||LFP full cells. Compared to other anodes in the Li full cells, the electrochemical performance of this ALi cluster anode performs significant advantages, especially more than the current research work on amorphous Li anode (Table S2, Supporting Information).

## 3 Conclusions

In conclusion, we have developed heteroatom‐activating electronegative sites and advanced, orderly, and robust SEI systems in the HDGs matrixes for regulating and stabilizing ALi clusters as an excellent Li anode. By the effective assistance of cryo‐TEM characterization, an ALi cluster anode with stable structure has been revealed. High surface area of 1764 m^2^ g^−1^ on the activating HDGs plating matrixes can effectively reduce the local current density, and showed an ultralow local current density of 4.72 × 10^−6^ mA ${\mathrm{cm}}_{\mathrm{HDGs}}^{-2}$ as Li deposition on HDGs plating matrixes. Heteroatom‐activating electronegative sites in the activating HDGs plating matrixes are acting as the lithiophilic electronegative sites to enhance electrostatic interaction of Li^+^ and activating surface. Hence, the activating HDGs plating matrixes can offer a larger binding energy and a low Li nucleation barrier with displaying small nucleation overpotentials of 13.9 and 10 mV at 0.1 mA cm^−2^ in the FEC‐ester and LiNO_3_‐ether electrolytes, respectively. An advanced, multilayer‐ordered, and robust SEI system comprised of inorganic‐rich components can increase the mechanical durability and offer fast Li^+^ diffusion to ensure highly reversible and more robust Li plating/stripping process. Over 2800‐long‐term cycles with ultrastable dendrite‐free cycling are achieved. Moreover, using LFP as the cathode, ALi clusters are also obtained on ALi‐HDGs‐N hosts. The LFP‐ALi‐HDGs‐N||LFP full cells exhibit highly initial specific capacities of 165.5 and 164.3 mAh g^−1^ at 0.1 C in FEC‐ester electrolytes and LiNO_3_‐ether electrolytes, respectively. The remarkable capacity retentions as high as 93% and 91% are maintained after 150 cycles at 0.2 C. In this paper, structure viability, highly electrochemical reversibility, and excellent performance in ALi cluster anodes are effectively regulated on the heteroatom‐activating electronegative sites and advanced, multilayer‐ordered, and robust SEI systems in the activating HDGs plating matrixes for ultrastable dendrite‐free cycling.

## 4 Experimental Section

### Syntheses of Three‐Dimensional Activating HDGs Matrixes with Heteroatom‐Activating Electronegative Sites

50 mL fluorinated epoxy resin dispersions (diphenolic‐based hexafluoropropane glycidyl ether resins, >99%, Shanghai Dongfu Chemical Technology Co., Ltd.) were well‐mixed with 20 g KOH for 6 h. Then, the obtained resin/KOH dispersions were dried at 80 °C for the viscous mixtures, and pyrolyzed at 900 °C for 2‐h activated etching in high‐purity Ar atmosphere. The heating rate during the pyrolysis was 3 °C min^−1^. Deionized water and 4 m HCl solution were used for washing several times to remove the impurities, and the collected samples were dried at 90 °C overnight. In the pyrolytic graphitization process, benefitted by the ethylenediamine curing agents, triethanolamine accelerators and epichlorohydrin intermediates contained in the fluorinated epoxy resins, the fluorine (F), oxygen (O), nitrogen (N), and chlorine (Cl) codoping graphene‐like films with the heteroatom‐activating electronegative sites (HDGs) were prepared.

### Electro‐Deposition of Amorphous‐Li Clusters on the Activating HDGs Matrixes

The as‐prepared HDGs materials and polyvinylidene fluoride (PVDF) binders with a mass ratio of 9:1 were stirred into the dispersed solvent of *N*‐methyl‐2‐pyrrolidone (NMP) for 4 h. Then, using a copper film as the substrate, the above dispersed slurry was uniformly coated as a 250 µm thick layer. After a vacuum heating at 90 °C for 12 h, the coated foils were cut into circular pieces with the diameter of 11 mm, and the loading weight of HDGs materials on each copper piece was ∼1.2 mg cm^−2^. To deposit ALi clusters on the activating HDGs matrixes at the certain current densities of 0.1, 0.2, 0.5, 1, and 2 mA cm^−2^, 2032‐type coin cell configuration with Li metal as the reference and counter electrode was worked and assembled in the glovebox that was filled with high purity Ar (H_2_O < 0.1 ppm, and O_2_ < 0.1 ppm). Celgard 2400 with a diameter of 16 mm, 1.2 m LiTFSI in mixed 1,2‐dimethoxyethane, and 1,3‐dioxolane (DME/DOL, 1:1 by volume) solution added with 0.2 m LiNO_3_ (LiNO_3_‐ether electrolytes), and 1.2 m LiPF_6_ in mixed ethylene carbonate and dimethyl carbonate (EC/DMC, 1:1 by volume) solution with 13 wt% FEC as additives (FEC‐ester electrolytes) were, respectively, employed for the separator, ether‐, and ester‐based electrolytes. The amounts of electrolytes were 40 µL. Electro‐deposition of amorphous Li clusters on the HDGs was performed on a Land CT2001A battery‐testing system. In the galvanostatic regime, the ALi clusters stabilizing on the activating HDGs matrixes (ALi‐HDGs) would nucleate and grow with the increase of electroplating time. As depositing on the activating HDGs matrixes at 0.1 mA cm^−2^ in nucleation and growing for 10, 20, and 30 min, the obtained samples were defined as ALi‐HDGs‐N, ALi‐HDGs‐10, ALi‐HDGs‐20, and ALi‐HDGs‐30.

### Materials Characterization

Raman spectra were recorded on a DXRxi Raman spectrometer with a 532 nm excitation wavelength. ASAP 2020 Accelerated Surface Area and Porosimetry System was used to measure the specific surface area and pores size distribution. XPS measurements were taken on an Axis Supra spectrometer (Kratos Analytical Ltd.). The microscopic observations were characterized by field‐emission scanning electron microscope (S4800, Hitachi), TEM (JEM‐2100F, JEOL) and spherical aberration‐corrected TEM (Titan Cubed Themis G2 300, FEI). Cryo‐TEM characterizations were performed on JEM‐2100F cryo‐TEM and operated at 200 kV, in which the ALi‐HDGs samples and cryo‐TEM test temperatures were kept at about −176 °C by the liquid nitrogen. ALi‐HDGs samples onto the cryo‐TEM holder were placed in the Ar‐filled glovebox, and then sealed by a shutter in the air‐insulated tube to prevent the air exposure. The schematic illustration of the details to prepare and transfer the samples into cryo‐TEM is shown in Figure S27, Supporting Information.

### Electrode Preparation and Electrochemical Measurements

Two ALi‐HDGs‐10 working electrodes with the amorphous structure in the same electroplating time were assembled as the symmetrical batteries in 2032‐type coin cell configuration. LFP, carbon black, and PVDF were mixed together by 85:5:10 in NMP solvent for 4 h. A 10 µm film with the above dispersed slurry on Al foil was formed and then dried at 120 °C for 12 h. The composite films were cut into 8‐mm pieces as the cathodes, and the LFP‐loading weight in each piece was ∼0.18 mg cm^−2^. In the full‐cell preparation, LFP cathodes and SEI stabilized ALi‐HDGs‐N anode worked. The activating HDGs matrixes coated as the 11‐mm pieces of 1.2 mg cm^−2^ were cycled at 0.1 mA cm^−2^ for ten cycles between 3 and 0.01 V for stabilizing SEI, and then, after Li nucleating on these SEI stabilized HDGs matrixes (ALi‐HDGs‐N) at 0.1 mA cm^−2^ (Figure S7, Supporting Information), the anodes were prepared and used in the LFP full cells. LFP‐ALi‐HDGs‐N||LFP full cells were evaluated by using 2032‐type coin cell that assembled in an Ar‐filled glovebox (H_2_O < 0.1 ppm, and O_2_ < 0.1 ppm, SG1200‐750TS, Vigor Gas Purification Tech. Co., Ltd.). The separator, LiNO_3_‐ether, and FEC‐ester electrolytes were the Celgard 2400 with a diameter of 16 mm, 1.2 m LiTFSI in mixed DME and DOL (DME/DOL, 1:1 by volume) solution added with 0.2 m LiNO_3_, and 1.2 m LiPF_6_ in mixed EC and DMC (EC/DMC, 1:1 by volume) solution with 13 wt% FEC as additives, respectively. The amounts of electrolytes were 40 µL. After charging to 3.8 V, Li depositions with amorphous states were still achieved and remained on the HDGs (LFP‐ALi‐HDGs‐N).

To verify the CEs of ALi clusters plating on the activating HDGs matrixes, SEI‐stabilized engineering was cycled at 0.1 mA cm^−2^ for ten cycles between 3 and 0.01 V. With pure Li metal as the reference electrode and counter electrode, and SEI‐stabilized HDGs matrixes as the working electrode, the CE determinations were tested at 0.1 mA cm^−2^ for 10 and 20 min, and the stripped voltage was 2 V. Galvanostatic charging/discharging measurements were obtained by a Land CT2001A battery‐testing system (Wuhan LAND Electronic Co., Ltd.). Cyclic voltammetry was carried out on an Autolab PG302N electrochemical workstation (Metrohm, Netherlands) with a scan rate of 0.1 mV s^−1^.

## Conflict of Interest

The authors declare no conflict of interest.

## Supporting information

Supporting Information

## Data Availability

The data that support the findings of this study are available from the corresponding author upon reasonable request.
