# Supplementary material for: Effectively Regulating More Robust Amorphous Li Clusters for Ultrastable Dendrite‐Free Cycling
Source: Adv Sci (Weinh). 2021 Aug 3;8(19):2101584. doi: 10.1002/advs.202101584 (PMC8498897; doi:10.1002/advs.202101584)
Supplement: Supplementary file 1 — Supporting Information [file ADVS-8-2101584-s001.pdf]

© 2021 Wiley-VCH GmbH

ADVANCED SCIENCE
Open Access

Supporting Information

for *Adv. Sci.,* DOI: 10.1002/advs.202101584

Effectively regulating more robust amorphous Li clusters for ultra-stable dendrite-free cycling

*Shizhi Huang, Junfeng Yang, Luxiang Ma, Jingyi Ding, Xusheng Wang, Chengyuan Peng, Binglu Zhao, Mengxiong Cao, Junrong Zheng, Xin-Xiang Zhang and Jitao Chen**

Supporting Information

Effectively regulating more robust amorphous Li clusters for ultra-stable dendrite-free cycling

*Shizhi Huang, Junfeng Yang, Luxiang Ma, Jingyi Ding, Xusheng Wang, Chengyuan Peng, Binglu Zhao, Mengxiong Cao, Junrong Zheng, Xin-Xiang Zhang and Jitao Chen**

S. Huang, J. Yang, Dr. L. Ma, J. Ding, C. Peng, Dr. B. Zhao, Dr. M. Cao, Prof. J. Zheng, Prof. X. -X. Zhang and Prof. J. Chen
Beijing National Laboratory for Molecular Sciences, College of Chemistry and Molecular Engineering
Peking University, Beijing 100871, China
E-mail: chenjitao@pku.edu.cn

Dr. X. Wang
Technical Institute of Physics and Chemistry
Chinese Academy of Sciences, Beijing 100190, China

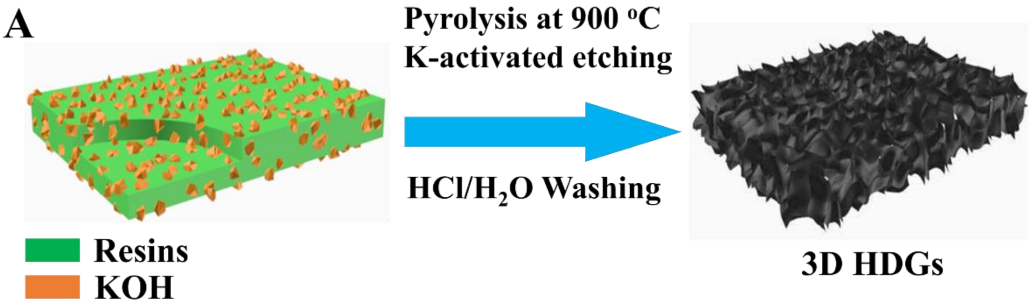
A
Pyrolysis at 900 °C
K-activated etching
HCl/H2O Washing
Resins
KOH
3D HDGs

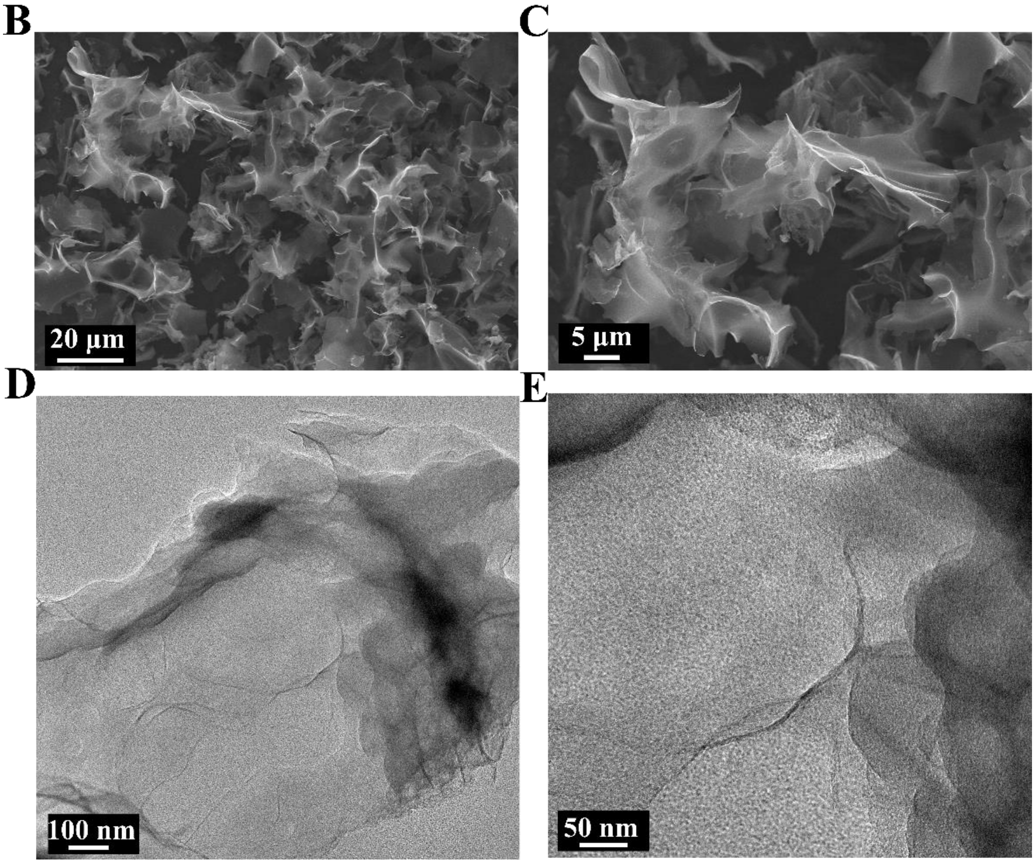

Figure S1. Preparation schematic illustration and morphology characterizations of the HDGs. (A) Schematic illustration of the HDGs preparation. Morphology characterizations show in SEM (B, C) and TEM (D, E) images.

During the pyrolysis process at 900 °C, three-dimensional heteroatom-doped graphene-like films (3D HDGs) were produced by the KOH activated etching, which according to the reactions as following:[1–3]

$$4KOH + C = K_2CO_3 + K_2O + 2H_2 \quad (S1)$$

$$K_2CO_3 + 2C = 2K + 3CO \quad (S2)$$

$$K_2O + C = 2K + CO \quad (S3)$$

$$CO + H_2O = CO_2 + H_2 \quad (S4)$$

$$4K + 3CO_2 = 2K_2CO_3 + C \quad (S5)$$

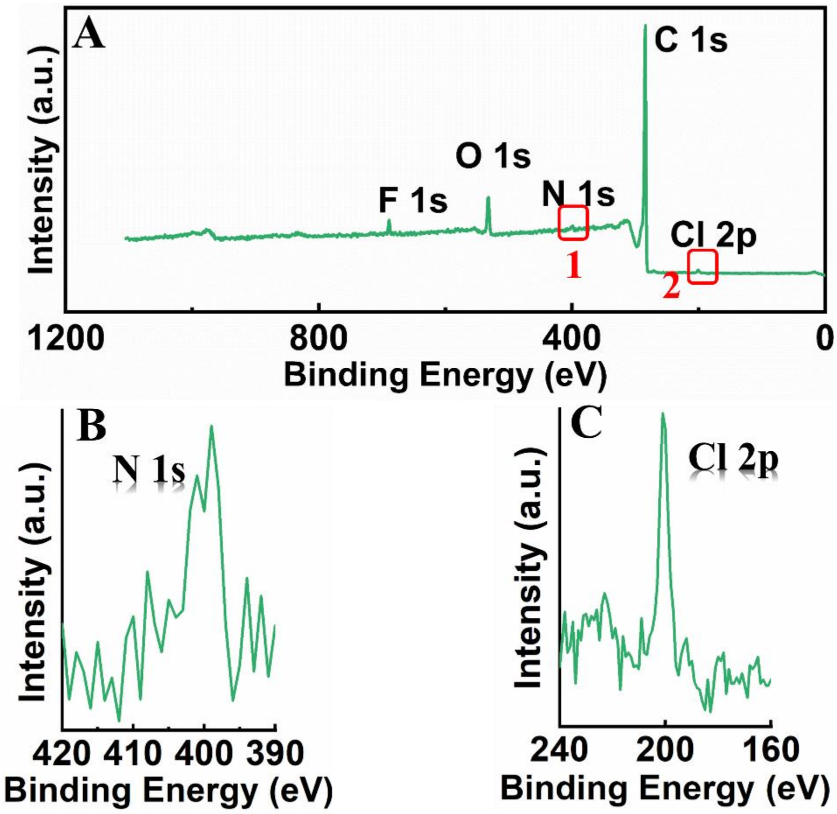

Figure S2. XPS survey spectra of the HDGs. (B) N 1s and (C) Cl 2p corresponding to the marked regions 1, 2 in (A).

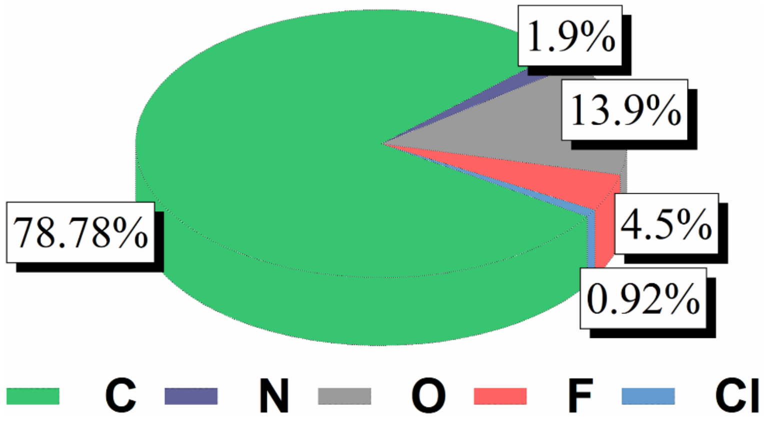

Figure S3. Surface atomic ratios of C, N, O, F, Cl in high-resolution XPS results.

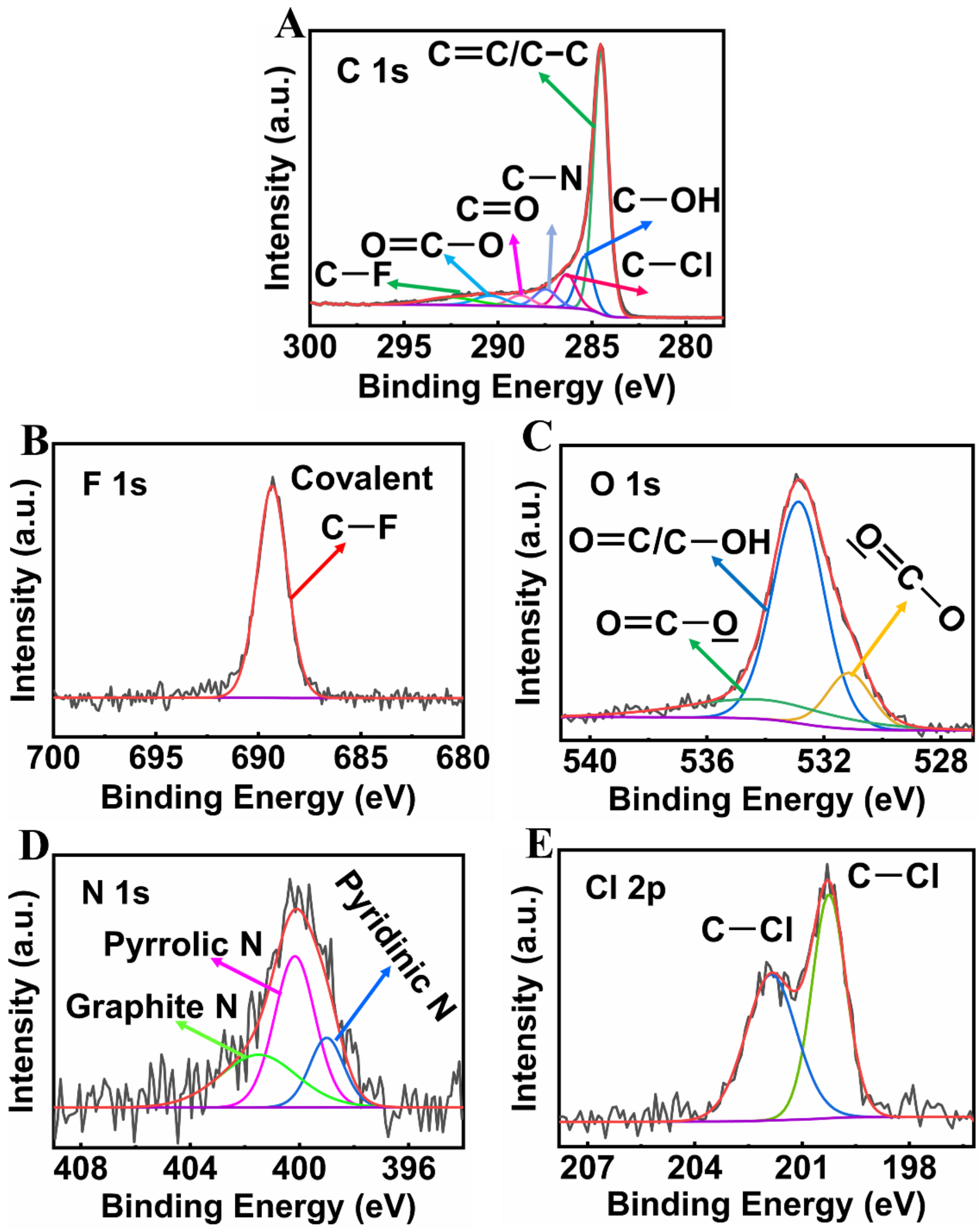

Figure S4. XPS spectra of C 1s, F 1s, O 1s, N 1s and Cl 2p.

F 1s XPS spectra exist in 689.3 eV, and implying the covalent C−F bond in HDGs. For O 1s, the peaks at 534.5, 532.8 and 531.1 eV are ascribed to the oxygen single bond of O=C−O in esters and carboxylic acids, O=C/C−OH (carbonyl and hydroxyl), and oxygen double bond of O=C−O in carboxyl group (COO−), respectively.[4–6] In N 1s fitting curves, the appeared peaks are at 401.5, 400.1 and 399 eV, which corresponding to the graphitic N, pyrrolic N and pyridinic N, respectively.[7] Two peaks at 201.8 and 200.2 eV are the binding energies of Cl−C in the Cl $2p_{1/2}$ and Cl $2p_{3/2}$.[8–9] In the meanwhile, the above fitting results are also consistent in C 1s.

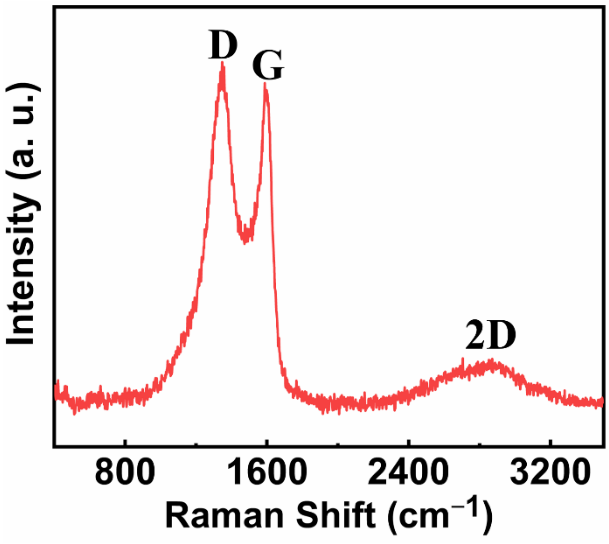

Figure S5. Raman spectra of HDGs.

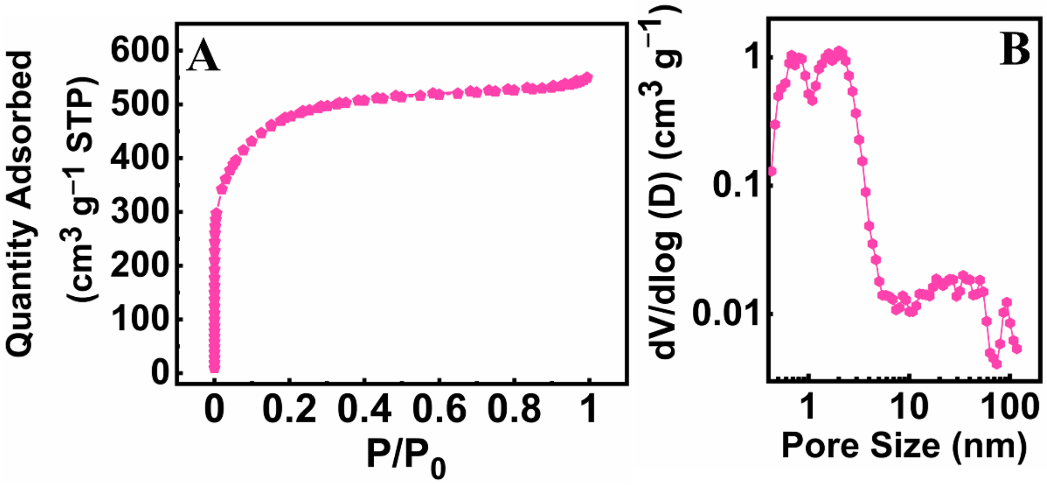

Figure S6. (A) N_2 adsorption-desorption isotherm and (B) pore size distribution curve of HDGs.

In the pore size distribution curve, the hierarchical porous structure including micro-, meso- and macro-pores was evaluated in HDGs. Based on the above Equation (S1-5), H_2, CO, CO_2, and vaporized H_2O positively act on the formation of hierarchical porosity through the gasification of carbon in the pyrolysis process.[1] Meanwhile, KOH activation, namely chemical etching, also contributes to the 3D hierarchical porous construction.

According to the classical heterogeneous nucleation theory, the critical nucleation radius (r^*) and its related thermodynamic equations are following:[10]

$$r^* = 2\gamma_{LE}/\Delta G_V \qquad \text{(S6)}$$

$$\Delta G^*_{het} = S(\theta)\,\Delta G^*_{hom} \qquad \text{(S7)}$$

$$S(\theta) = (2 + \cos\theta)(1 - \cos\theta)^2/4 \qquad \text{(S8)}$$

$$\cos\theta = (\gamma_{GE} - \gamma_{LG})/\gamma_{LE} \qquad \text{(S9)}$$

In these equations, γ_{LE}, γ_{GE} and γ_{LG} are the interfacial free energy of Li/electrolyte, HDGs plating matrixes/electrolyte, and Li/HDGs plating matrixes, respectively. ΔG_V, ΔG^*_{het}, and ΔG^*_{hom} are ascribed to the free energy change of Li from electrolyte to anode, heterogeneous and homogeneous nucleation barrier, respectively. Heteroatom-activating sites can enhance the binding energy between Li and HDGs plating matrixes, where γ_{LG} would reduce. As a result, the θ is decreased, and the volume needed of Li-nucleation in reaching r^* is also reduced, which is evident in support of stably achieving amorphous-Li phase by aggregating a small amount of Li atoms for a reduced volume.

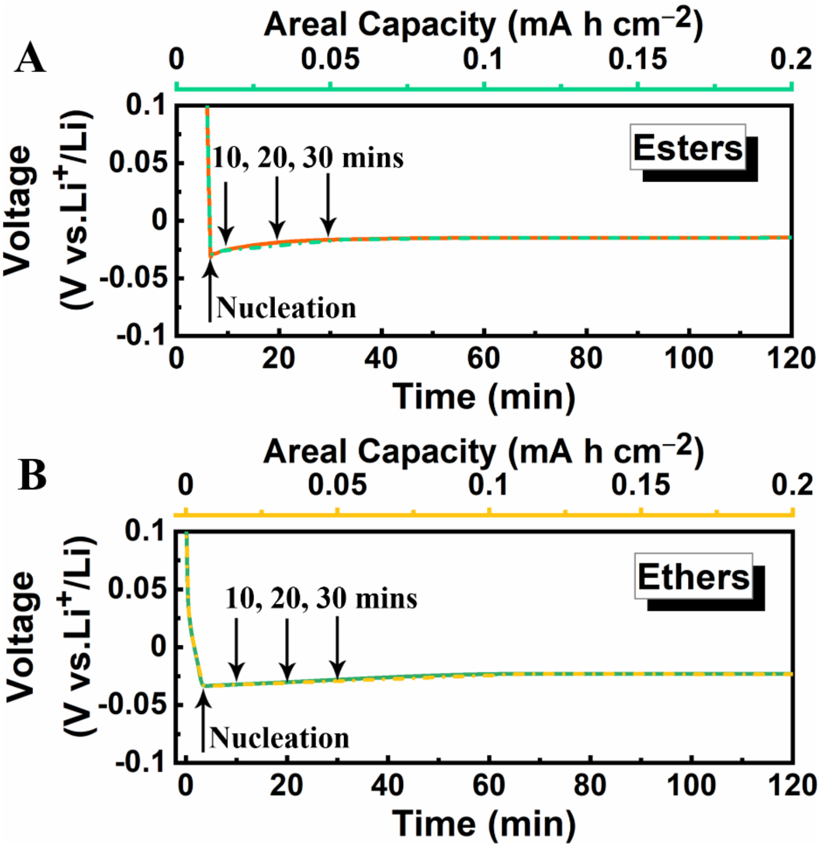

Figure S7. The deposition curves of ALi nucleation and growth on the activating HDGs plating matrixes at 0.1 mA cm^{-2}. The electrolytes are (A) FEC-ester and (B) $LiNO_3$-ether electrolytes.

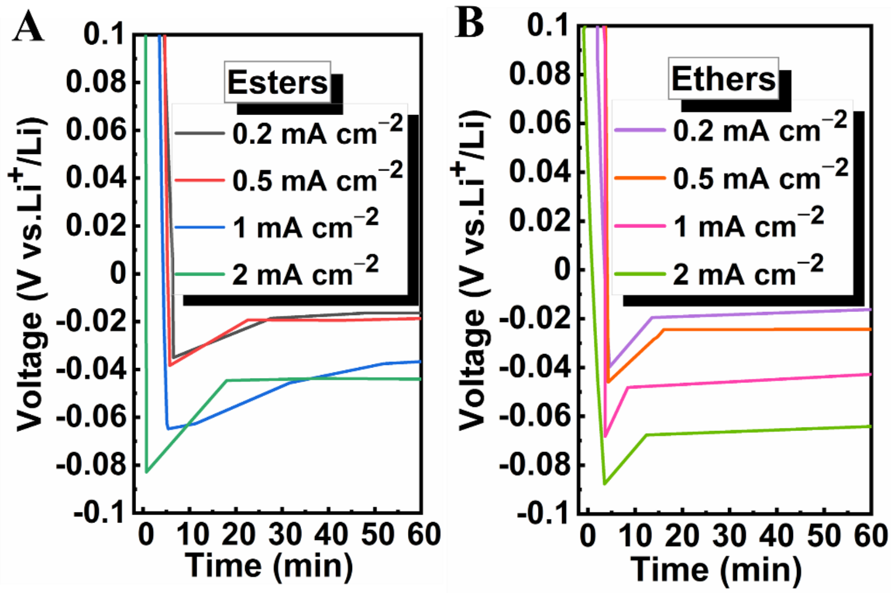

Figure S8. The voltage-time curves of Li nucleation and growth on the activating HDGs plating matrixes at 0.2, 0.5, 1 and 2 mA cm^{-2}. The electrolytes are (A) FEC-ester and (B) $LiNO_3$-ether electrolytes.

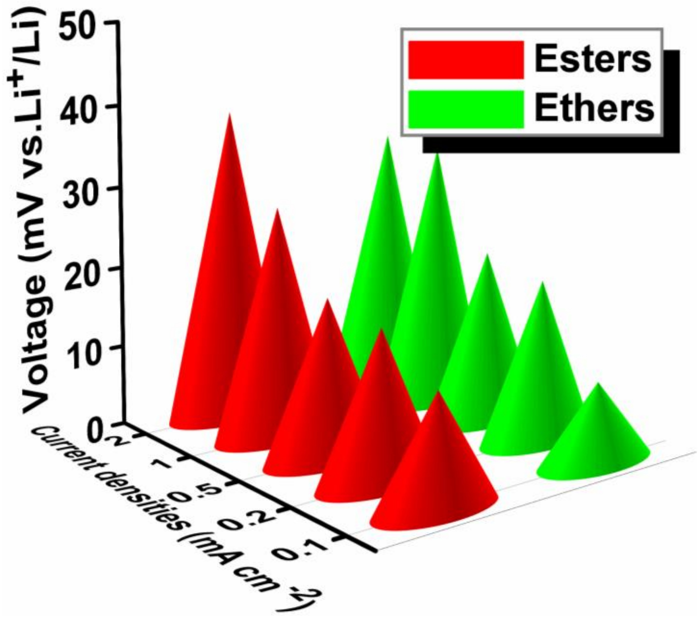

Figure S9. Nucleation overpotentials of Li-deposition on the activating HDGs plating matrixes.

There is no particularly noticeable change in the nucleation overpotentials when Li-deposition current densities are increased to 0.2, 0.5 mA cm^{-2}, either in FEC-ester electrolytes or in $LiNO_3$-ether electrolytes. Even enlarging 20 times to 2 mA cm^{-2}, the nucleation overpotential is only increased to 2.7 times, which showing a larger binding energy and excellent lithiophilicity between ALi clusters and activating HDGs plating matrixes that composed of heteroatom-activating electronegative sites.

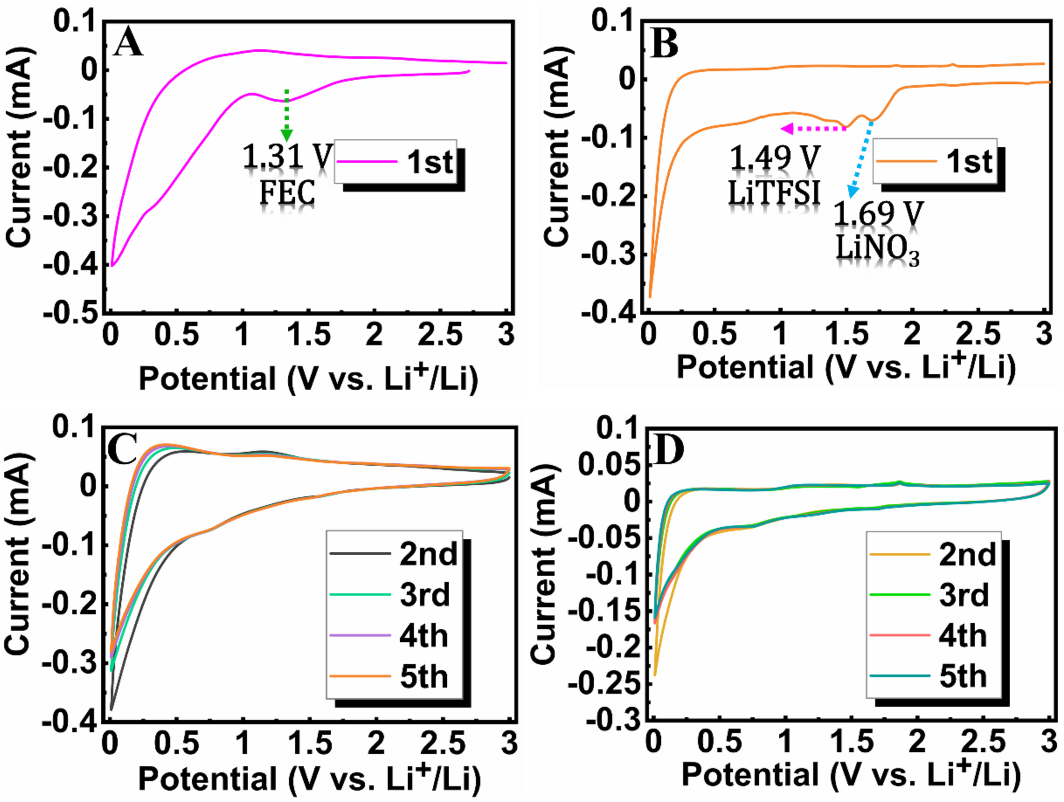

Figure S10. Cyclic voltammetry curves between 0.01−3 V at a scanning rate of 0.1 mV s^{-1}. The electrolytes are (A–C) FEC-ester and (B–D) $LiNO_3$-ether electrolytes.

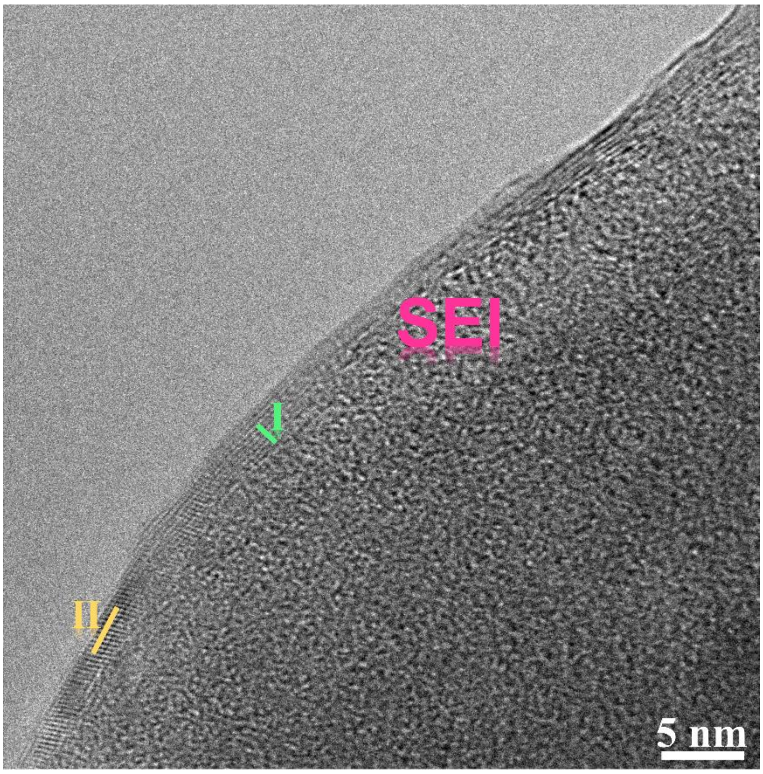

Figure S11. Original high-resolution cryo-TEM image of SEI in Figure 4A.

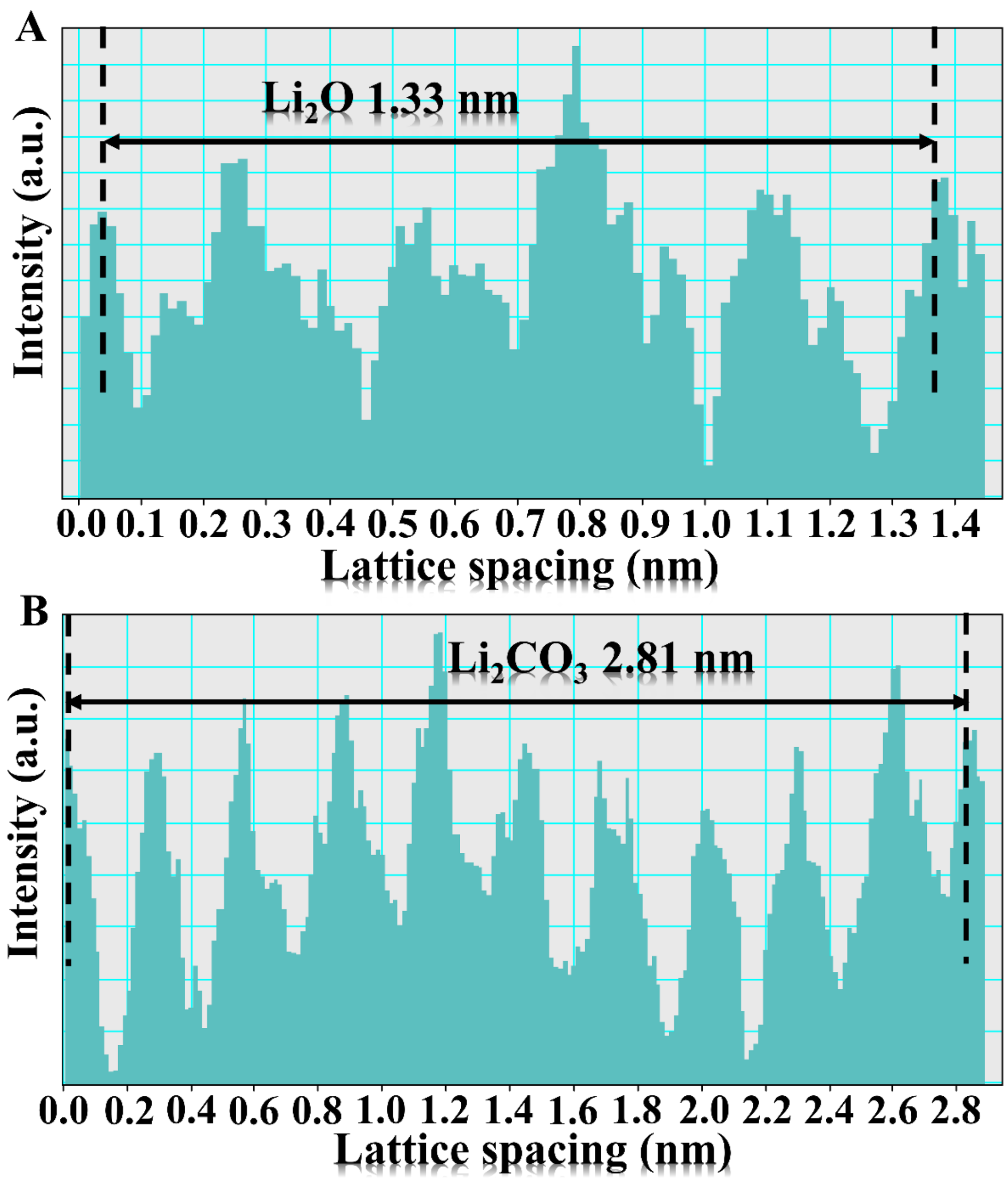

Figure S12. Integrated pixel intensities of Li_2O and Li_2CO_3 lattices. (A) and (B) corresponded to the areas I, II in Figure S11.

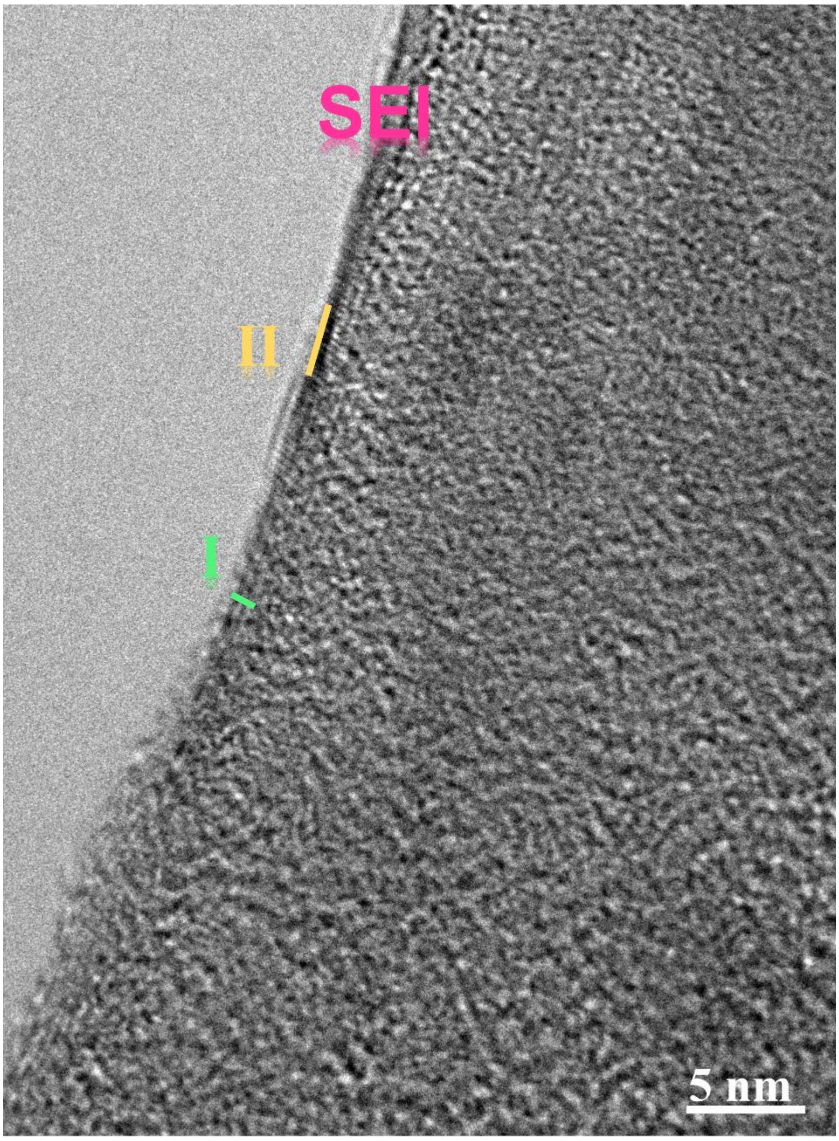

Figure S13. Original high-resolution cryo-TEM image of SEI in Figure 4C.

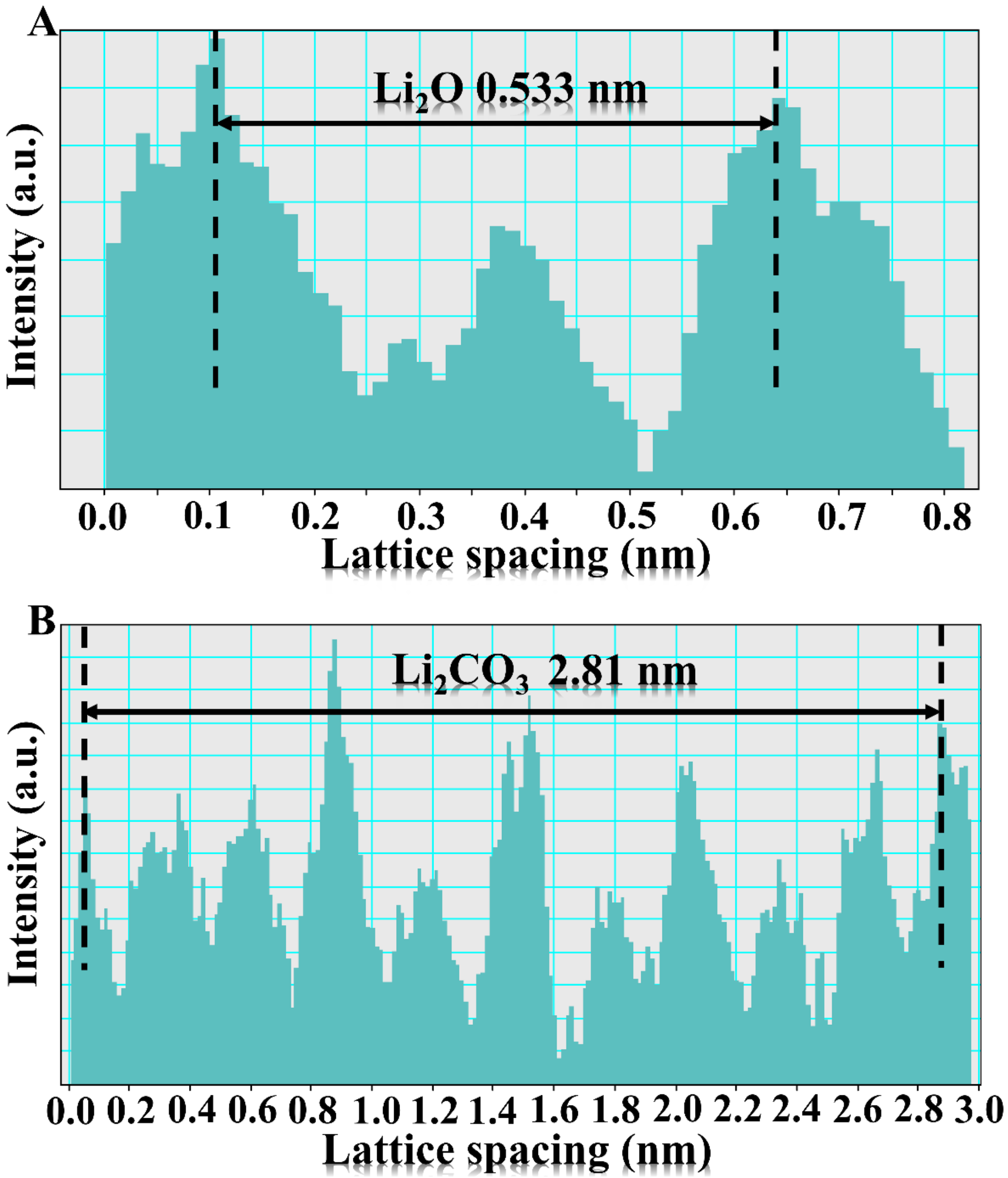

Figure S14. Integrated pixel intensities of Li_2O and Li_2CO_3 lattices. (A) and (B) corresponded to the areas I, II in Figure S13.

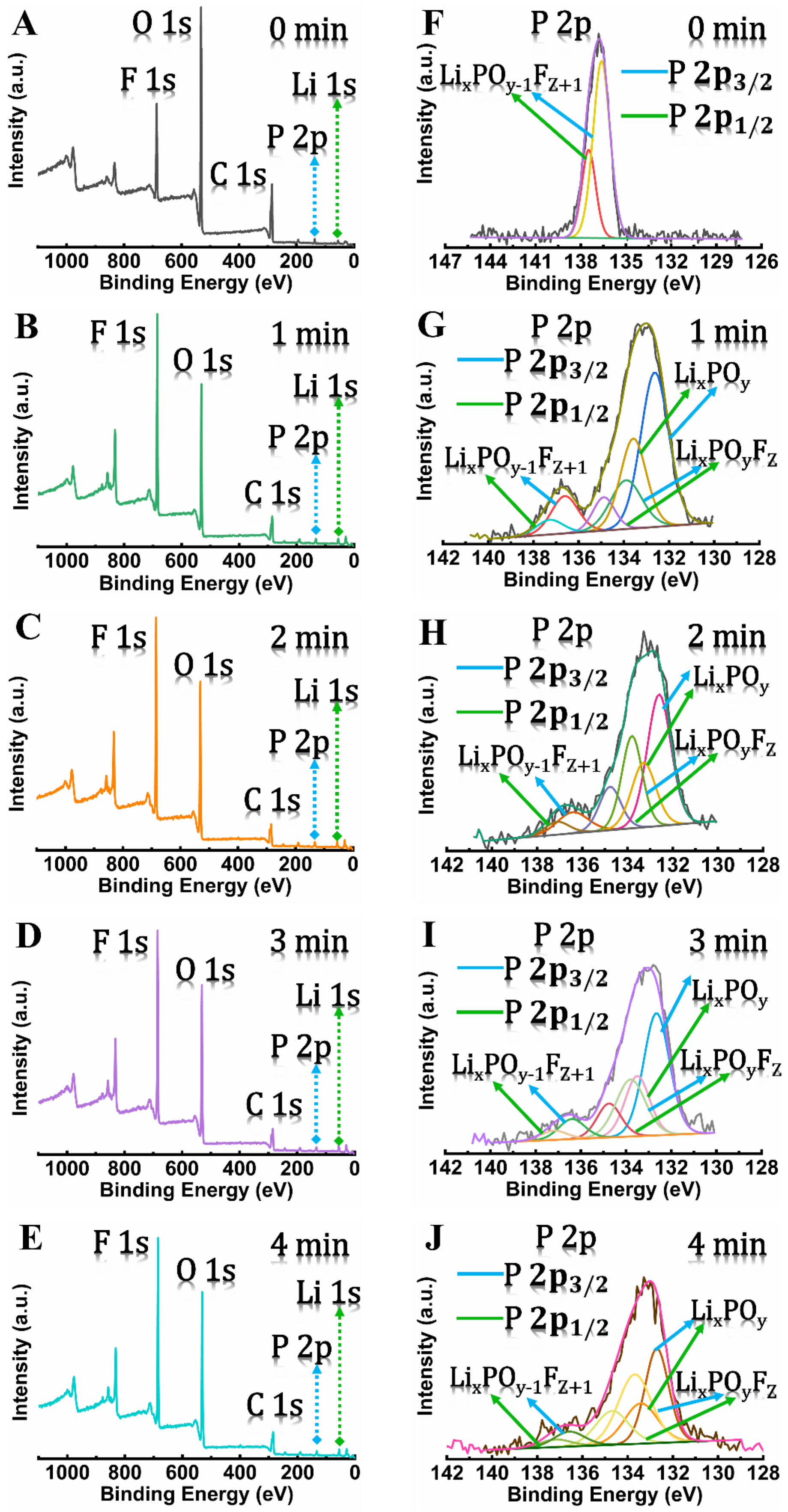

Figure S15. In-depth XPS survey and P 2p spectra. (A and F) 0 min, (B and G) 1 min, (C and H) 2 min, (D and I) 3 min, and (E and J) 4 min in FEC-ester electrolytes.

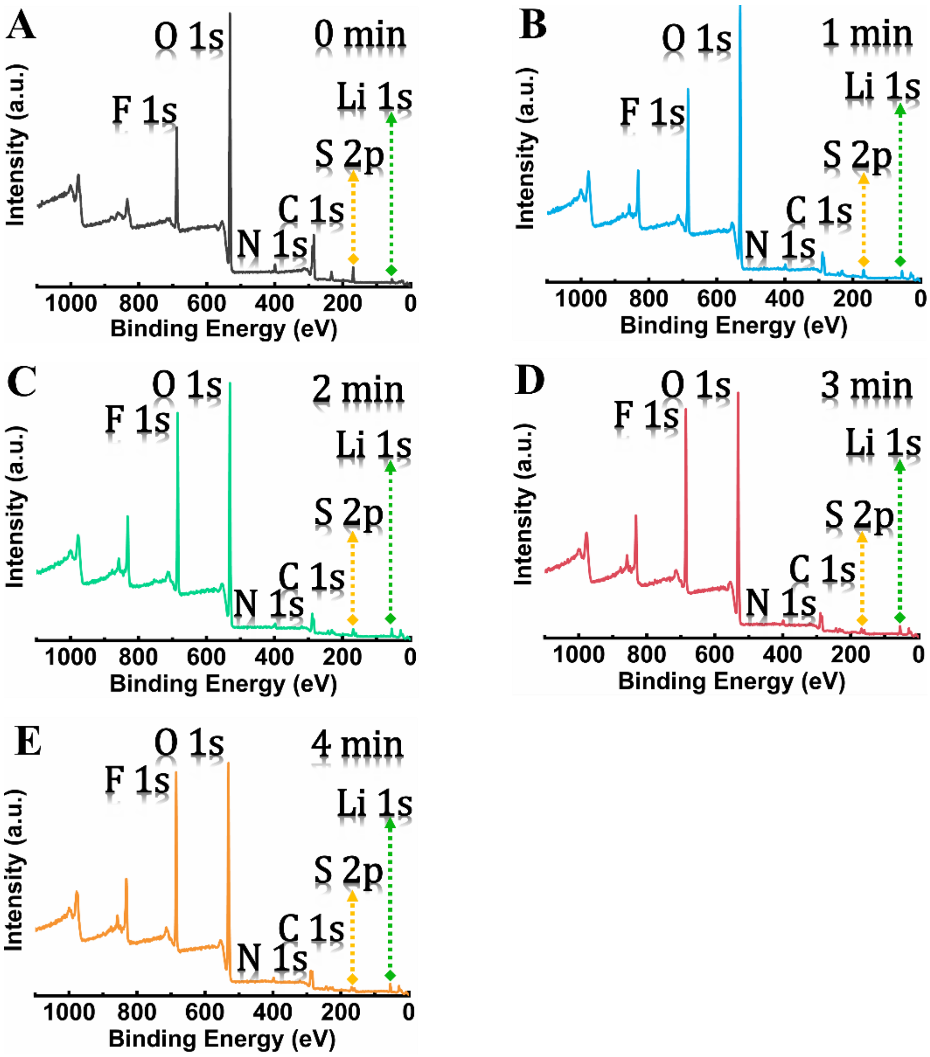

Figure S16. In-depth XPS survey spectra. (A) 0 min, (B) 1 min, (C) 2 min, (D) 3 min, and (E) 4 min in $LiNO_3$-ether electrolytes.

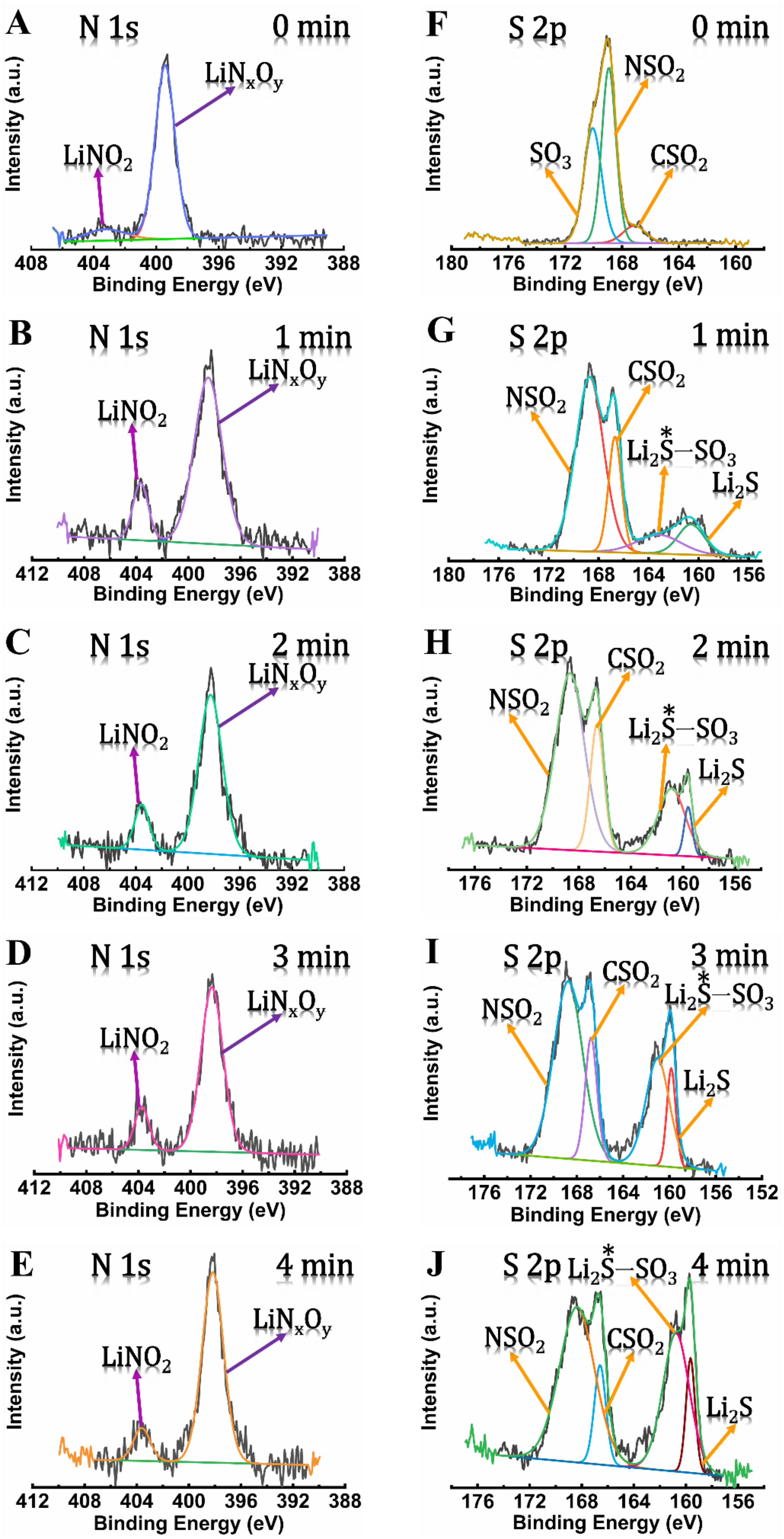

Figure S17. In-depth N 1s and S 2p spectra. (A and F) 0 min, (B and G) 1 min, (C and H) 2 min, (D and I) 3 min, and (E and J) 4 min in $LiNO_3$-ether electrolytes.

In Figure S17, the in-depth N 1s and S 2p XPS spectra are measured by the Ar^+ sputtering for 0, 1, 2, 3 and 4 min, that is ascribed to the SEI characterization in $LiNO_3$-ether electrolytes. These measured XPS spectra are fitted in CasaXPS program. Firstly, the background correction is used the Tougaard-type function and the detailed curve fittings are obtained by Gaussian-Lorentzian line shape.[11, 12] Besides, the absolute scale is adjusted to the non-oxidized carbon contribution of adventitious carbon (C_mH_n) in the C 1s spectra at 284.6 eV. In Figure S17, as the N 1s XPS spectra fitting, after the background correction and absolute scale adjusting, the two appeared peaks are fitted individually that are determined by absolute peak positions. As a result, the fitted peaks are observed at 399.4 and 403.3 eV that are confirmed as the LiN_xO_y species and $LiNO_2$ from $LiNO_3$ de-composition. Moreover, these fitting results are similar to the literature researched values that obtained and analyzed in the similar systems in the SEI formation by $LiNO_3$. Meawhile, the S 2p XPS spectra fittings are performed by these similar processes, and the 2p type peaks in the S 2p are also determined by absolute peak positions. In the S 2p fitting results, the peaks appeared at 168.7, 166.7, 163.2 and 160.6 eV are corresponded to the SO_3, NSO_2, CSO_2, $Li_2S{-}SO_3$ and Li_2S from LiTFSI decomposition.[13, 14]

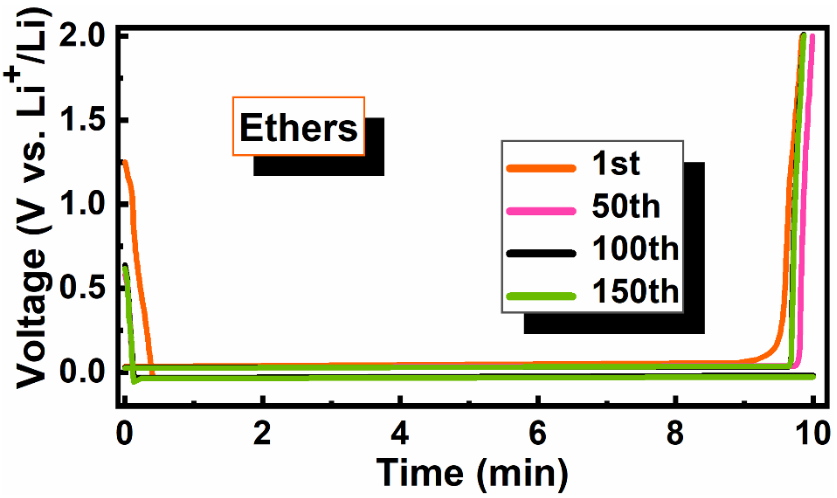

Figure S18. Voltage-time profiles of Li plating/stripping at 0.1 mA cm^{-2} for 10 min in $LiNO_3$-ether electrolytes.

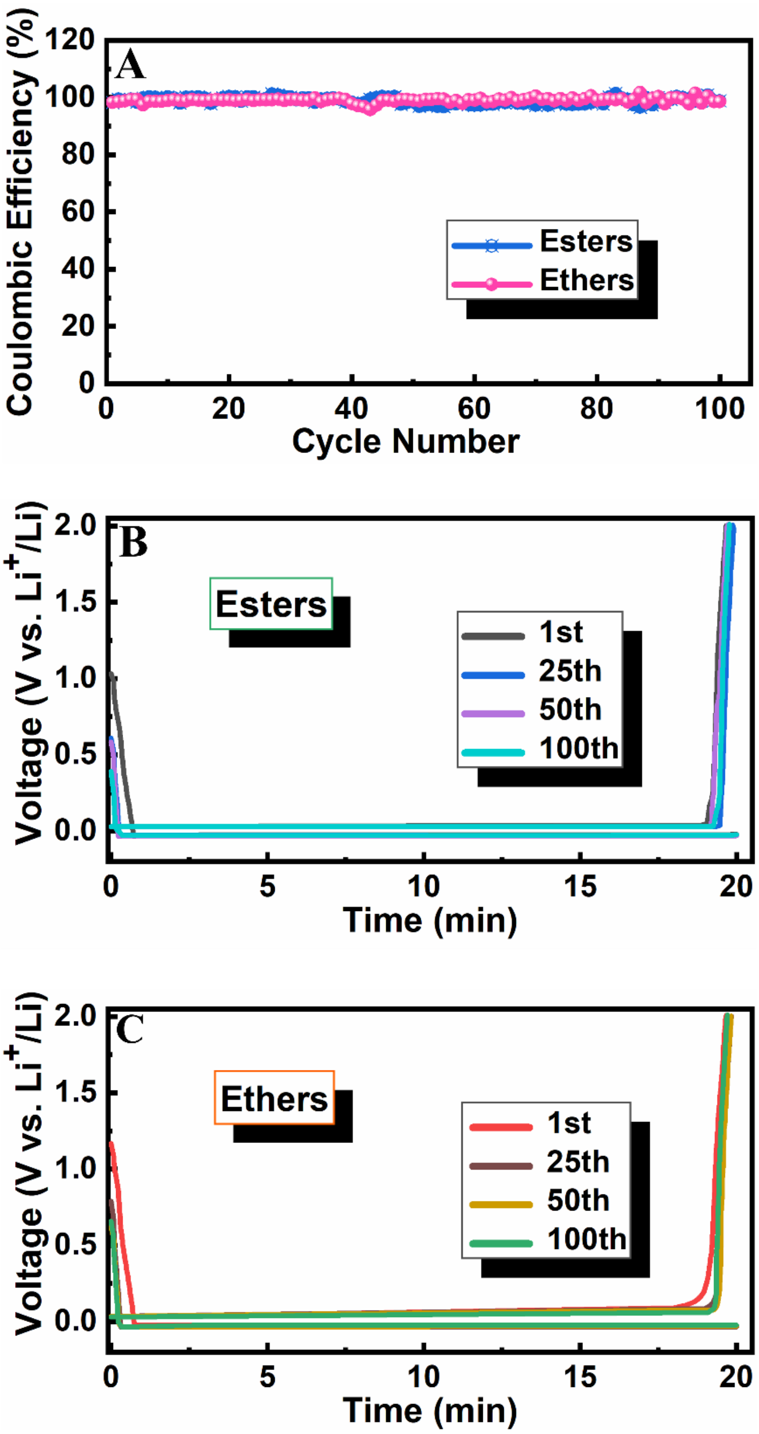

Figure S19. (A) Coulombic efficiencies and (B and C) voltage-time profiles of Li plating/stripping at 0.1 mA cm^{-2} for 20 min in FEC-ester electrolytes and $LiNO_3$-ether electrolytes.

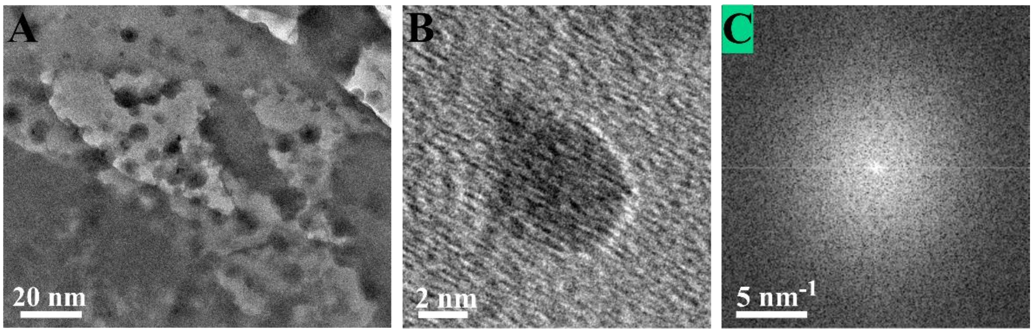

Figure S20. Structure viability of ALi anodes after 1000 plating/stripping cycles in FEC-ester electrolytes. (A–B) Cryo-TEM images of ALi-HDGs-10 and (C) corresponding FFT patterns of (B) in the ALi-HDGs-10 symmetrical cell that plating/stripping at 0.1 mA cm^{-2} for 10 min in each cycle.

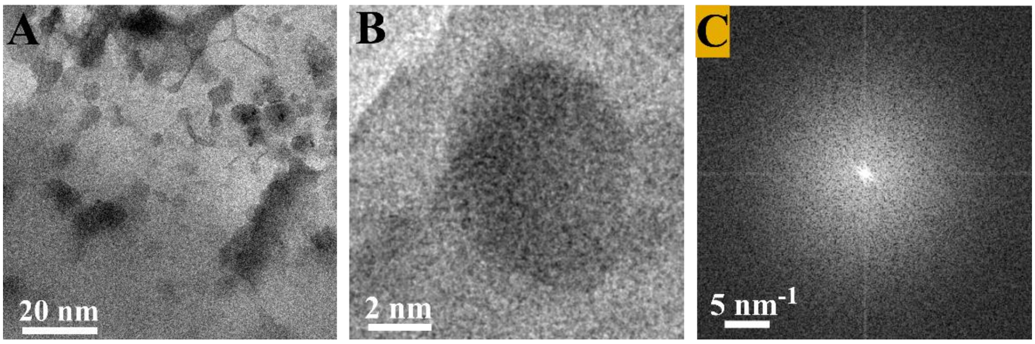

Figure S21. Structure viability of ALi anodes after 1000 plating/stripping cycles in $LiNO_3$-ester electrolytes. (A–B) Cryo-TEM images of ALi-HDGs-10 and (C) corresponding FFT patterns of (B) in the ALi-HDGs-10 symmetrical cell that plating/stripping at 0.1 mA cm^{-2} for 10 min in each cycle.

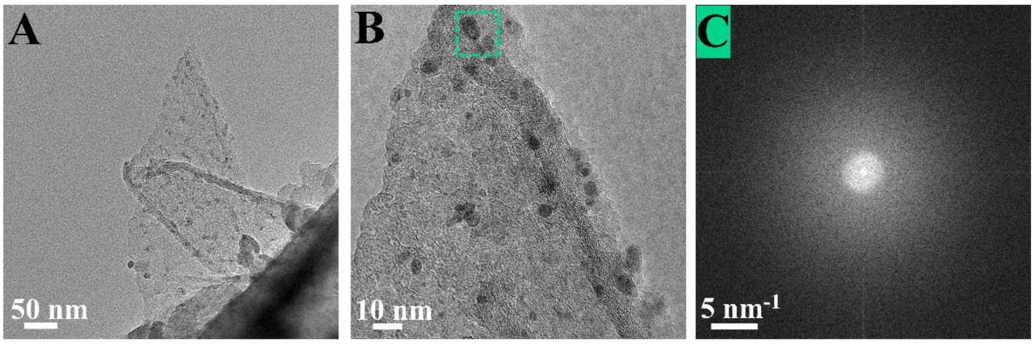

Figure S22. Amorphous-Li nanostructure grown on ALi-HDGs-N hosts from LFP cathode after charging at 0.1 C in FEC-ester electrolytes. (A–B) Cryo-TEM images and (C) corresponding FFT pattern of the marked area in (B).

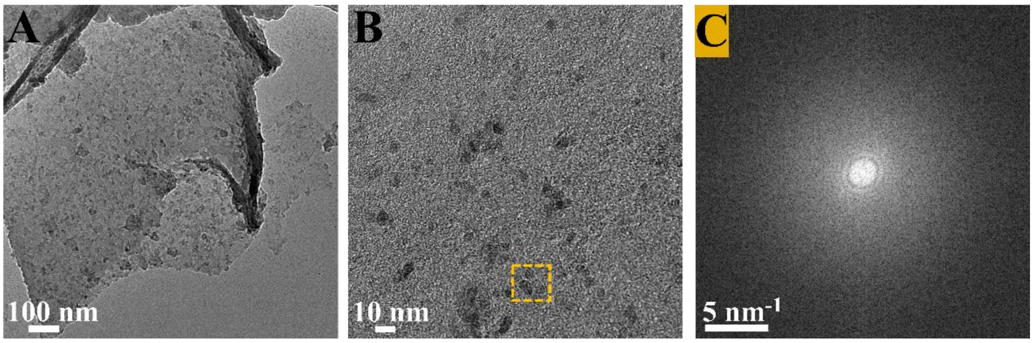

Figure S23. Amorphous-Li nanostructure grown on ALi-HDGs-N hosts from LFP cathode after charging at 0.1 C in $LiNO_3$-ether electrolytes. (A–B) Cryo-TEM images and (C) corresponding FFT pattern of the marked area in (B).

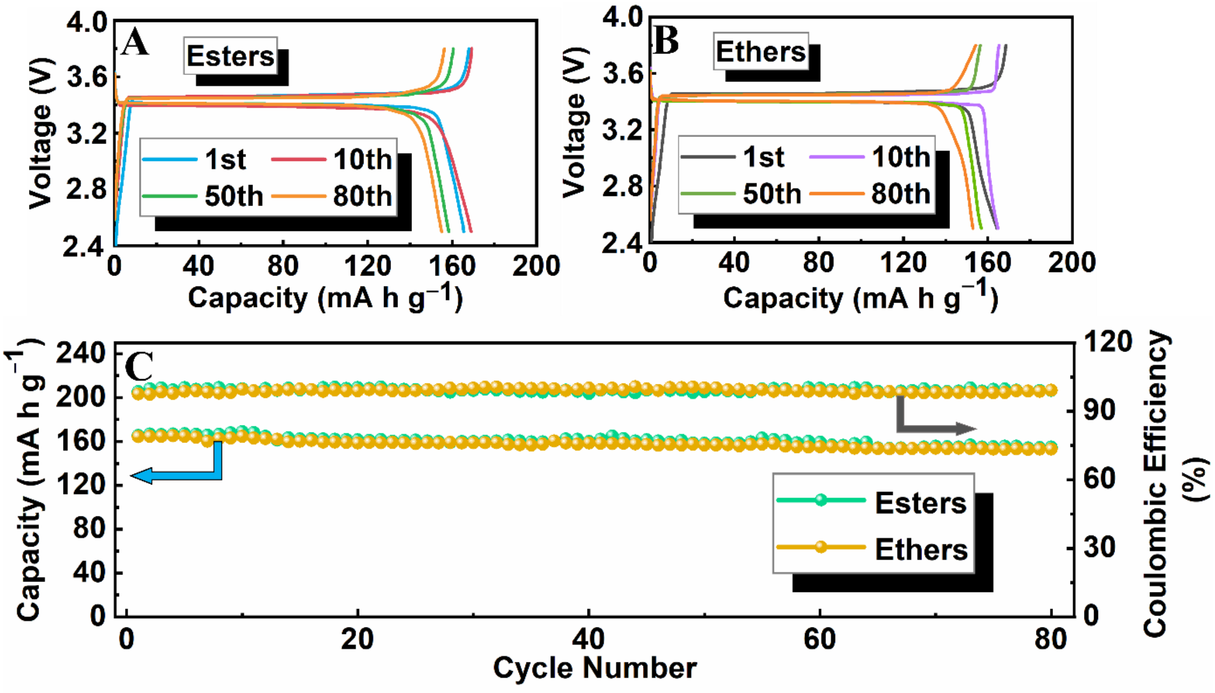

Figure S24. Electrochemical performance of LFP-ALi-HDGs-N||$LiFePO_4$ (LFP) full cells at 0.1 C in FEC-ester electrolytes and $LiNO_3$-ether electrolytes. (A–B) Charging/discharging profiles and (C) their corresponding cycling properties.

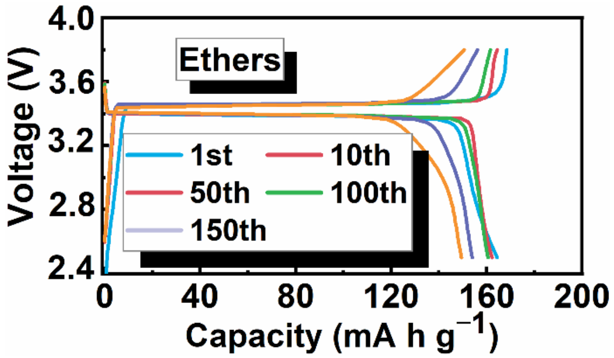

Figure S25. Charging/discharging profiles of LFP-ALi-HDGs-N||$LiFePO_4$ (LFP) full cells at 0.2 C in $LiNO_3$-ether electrolytes.

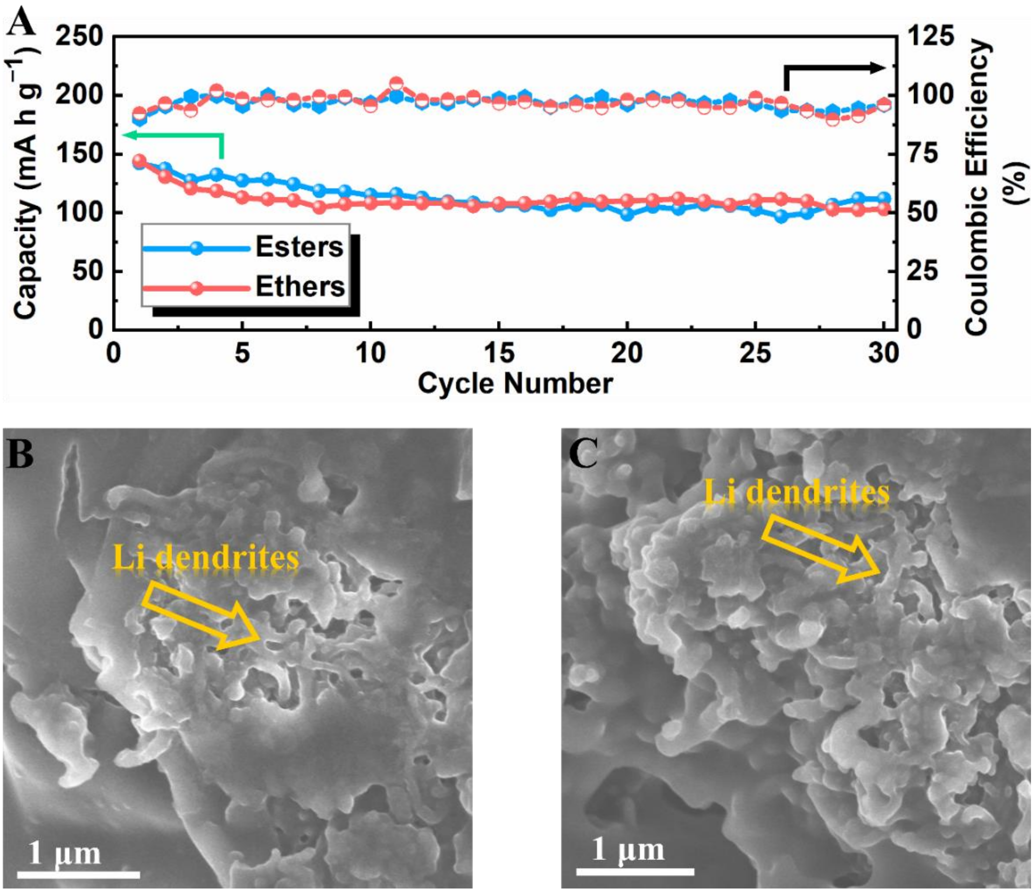

Figure S26. Electrochemical performances and Li morphologies on the HDGs hosts of a high weight loading LFP cathode. (A) The LFP-ALi-HDGs-N||$LiFePO_4$ (LFP) full cells were cycled at 0.2 C in FEC-ester electrolytes and $LiNO_3$-ether electrolytes. SEM images of Li on the HDGs hosts after the 31st charging at 0.2 C in the (B) FEC-ester electrolytes and (C) $LiNO_3$-ether electrolytes. The weight loading of LFP cathode is ~8.05 mg cm^{-2}.

A high weight loading LFP cathode of ~8.05 mg cm^{-2} is also used in the ALi-HDGs-N||$LiFePO_4$ (LFP) full cells. As shown in Figure S26A, during the 30-time charging/discharging cycles at 0.2 C, these full-cell performances continue to decay. The large capacity decays of 21.3% and 28.4% are obtained after 30 cycles in FEC-ester electrolytes and $LiNO_3$-ether electrolytes. The specific deposition states of Li growing on the HDGs hosts from LFP are revealed in Figure S26B and C. After the 31st charging at 0.2 C, the Li with dendrite growths are achieved and remained on the HDGs host. These appeared Li-dendrite growths can result in rapid capacity decays and low Coulombic efficiencies.

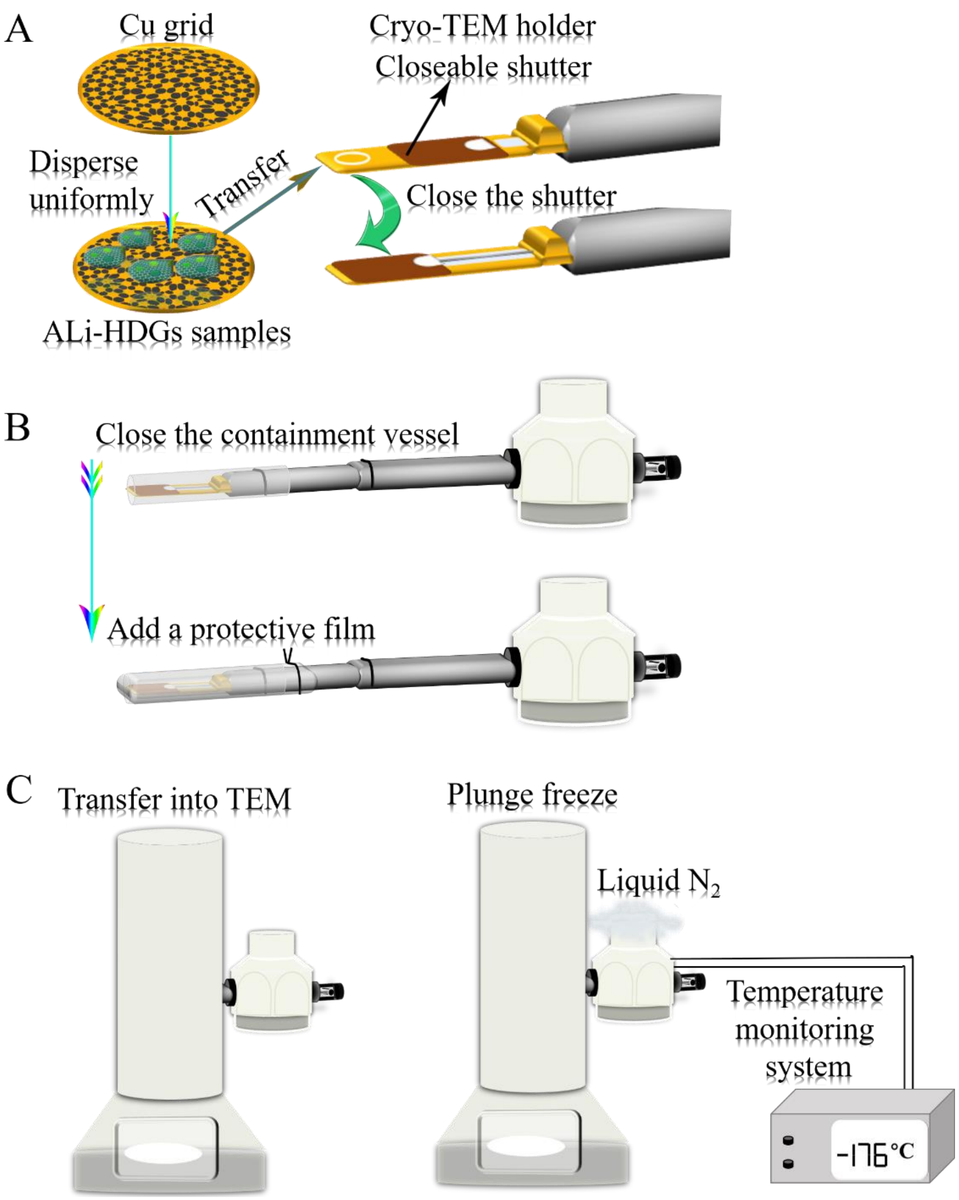

Figure S27. Schematic illustration of the details to prepare and transfer the samples into cryo-TEM.

In Figure S27A, the deposited ALi-HDGs samples are dispersed uniformly on a Cu grid, and then transferred and placed to the cryo-TEM holder. The closeable shutter near the placed position of Cu grid and a containment vessel as the air-insulated tube that filled with high purity Ar are closed (Figure S27 A and B). Moreover, we add a protective film to further promote the transfer of reliability. These sample preparations are completed in the glovebox that filled with high purity Ar (H_2O<0.1 ppm, and O_2<0.1 ppm). Next, the cryo-TEM holder with the ALi-HDGs loading is transferred to the transition chamber and keep it in a high vacuum state. Liquid N_2 is added and then the temperature is stable at −176 °C after 30 minutes. At the same time, the ALi-HDGs samples are also frozen to the same temperature with the assistance of liquid N_2 through the cryo-TEM holder in the high vacuum state (Figure

S27 C). After that, the ALi-HDGs micrographs are recorded and the cryo-TEM characterizations are operated at 200 kV with an electron dose rate of ~25 *e* $Å^{-2}$ S^{-1} for < 30 s.

Table S1. A summary of binding energy between doping heteroatoms and a Li atom in the conductive carbonaceous materials. These data results are from previous reports and conducted on first principles calculations.[10, 15–17]

Types of doping species	Binding energy (eV)
pyridinic N	−4.26
pyrrolic N	−4.46
graphitic N	−3.46
carboxylic group O	−2.86
ketone group O	−2.35
hydroxyl group O	−1.91
F	−0.83
Cl	−0.48

Table S2. Electrochemical performance comparisons between this ALi anodes and other anodes in the Li full cells.

Cathode material	Anode material	Current density	Capacity retention	Cycle number	Reference
NMC811	Li foil	0.5 C	<75%	80	[13]
NMC811	Li chip	0.5 C	48.2%	200	[14]
$LiFePO_4$	Porous Cu current-Li	0.5 C	90%	100	[18]
$LiFePO_4$	Ag-doped MOF-Li	0.5 C	87%	150	[19]
NMC811	Mo_2N@CNF-Li	0.3 C	90%	150	[20]
NMC811	Li metal	0.5 C	20%	200	[21]
NMC811	Li metal	0.5 C	50%	300	[21]
NMC622	Li foil	0.2 mA cm^{-2}	~52%	100	[22]
NMC622	Li foil	0.2 mA cm^{-2}	~86%	100	[22]
NMC622	Cu	C/3	56.9	50	[23]
NMC333	amorphous Li (~76.8%)	C/3	59.2%	50	[24]
**$LiFePO_4$**	**amorphous Li (100%)**	**0.2 C**	**93%**	**150**	**This work**
**$LiFePO_4$**	**amorphous Li (100%)**	**0.2 C**	**91%**	**150**	**This work**

References

[1] J. Wang and S. Kaskel, *J. Mater. Chem.* **2012**, *22*, 23710.
[2] Z. Y. Yang, Y. H. Wang, Z. Dai, Z. W. Lu, X. Y. Gu, H. Zhao, G. Z. Sun, W. Lan, Z. X. Zhang, X. J. Pan, J. Y. Zhou and E. Q. Xie, *Carbon* **2019**, *146*, 610.
[3] R. L. Zornitta, K. M. Barcelos, F. G. E. Nogueira and L. A. M. Ruotolo, *Carbon* **2020**, *156*, 346.
[4] H. Zhong, J. Wang, Y. Zhang, W. Xu, W. Xing, D. Xu, Y. Zhang, and X. Zhang, *Angew. Chem. Int. Ed.* **2014**, *53*, 14235.
[5] Q. Feng, S. Zhao, Q. Xu, W. Chen, S. Tian, Y. Wang, W. Yan, J. Luo, D. Wang and Y. Li, *Adv. Mater.* **2019**, *31*, e1901024.
[6] Y. Tang, L. Zhang, J. Chen, H. Sun, T. Yang, Q. Liu, Q. Huang, T. Zhu and J. Huang, *Energy Environ. Sci.* **2021**, *14*, 602.
[7] Z. S. Wu, W. Ren, L. Xu, F. Li and H. M. Cheng, *ACS Nano* **2011**, *5*, 5463.
[8] D. -W. Wang, K. -H. Wu, I. R. Gentle and G. Q. Lu, *Carbon* **2012**, *50*, 3333.
[9] J. Y. Kim, W. H. Lee, J. W. Suk, J. R. Potts, H. Chou, I. N. Kholmanov, R. D. Piner, J. Lee, D. Akinwande and R. S. Ruoff, *Adv. Mater.* **2013**, *25*, 2308.
[10] X. Chen, X. -R. Chen, T. -Z. Hou, B. -Q. Li, X. -B. Cheng, R. Zhang, Q. Zhang, *Sci. Adv.* **2018**, *5*, eaau7728.
[11] N. Schulz, R. Hausbrand, C. Wittich, L. Dimesso, and W. Jaegermann, *J. Electrochem. Soc.* **2018**, *165*, A833.
[12] N. Schulz, R. Hausbrand, L. Dimesso, and W. Jaegermann, *J. Electrochem. Soc.* **2018**, *165*, A819.
[13] J. Fu, X. Ji, J. Chen, L. Chen, X. Fan, D. Mu and C. Wang, *Angew. Chem. Int. Ed.* **2020**, *59*, 22194.
[14] S. Liu, X. Ji, N. Piao, J. Chen, N. Eidson, J. Xu, P. Wang, L. Chen, J. Zhang, T. Deng, S. Hou, T. Jin, H. Wan, J. Li, J. Tu and C. Wang, *Angew. Chem. Int. Ed.* **2021**, *60*, 3661.
[15] C. Ma, X. Shao and D. Cao, *J. Mater. Chem.* **2012**, *22*, 8911.
[16] R. Zhang, X. R. Chen, X. Chen, X. B. Cheng, X. Q. Zhang, C. Yan and Q. Zhang, *Angew. Chem. Int. Ed.* **2017**, *56*, 7764.
[17] H. Zhu, X. Gan, A. McCreary, R. Lv, Z. Lin and M. Terrones, *Nano Today* **2020**, *30*, 100829.
[18] S. H. Wang, Y. X. Yin, T. T. Zuo, W. Dong, J. Y. Li, J. L. Shi, C. H. Zhang, N. W. Li, C. J. Li and Y. G. Guo, *Adv. Mater.* **2017**, *29*, 1703729.
[19] S. Yuan, J. L. Bao, C. Li, Y. Xia, D. G. Truhlar and Y. Wang, *ACS Appl. Mater. Interfaces* **2019**, *11*, 10616.
[20] L. Luo, J. Li, H. Yaghoobnejad Asl and A. Manthiram, *Adv. Mater.* **2019**, *31*, e1904537.
[21] X. Fan, L. Chen, O. Borodin, X. Ji, J. Chen, S. Hou, T. Deng, J. Zheng, C. Yang, S. C. Liou, K. Amine, K. Xu and C. Wang, *Nat. Nanotechnol.* **2018**, *13*, 715.
[22] X. Fan, L. Chen, X. Ji, T. Deng, S. Hou, J. Chen, J. Zheng, F. Wang, J. Jiang, K. Xu and C. Wang, *Chem* **2018**, *4*, 174.
[23] J. Alvarado, M. A. Schroeder, T. P. Pollard, X. Wang, J. Z. Lee, M. Zhang, T. Wynn, M. Ding, O. Borodin, Y. S. Meng and K. Xu, *Energy Environ. Sci.* **2019**, *12*, 780.
[24] X. Wang, G. Pawar, Y. Li, X. Ren, M. Zhang, B. Lu, A. Banerjee, P. Liu, E. J. Dufek, J. G. Zhang, J. Xiao, J. Liu, Y. S. Meng and B. Liaw, *Nat. Mater.* **2020**, *19*, 1339.
